# Hsp90-stabilized MIF supports tumor progression via macrophage recruitment and angiogenesis in colorectal cancer

**DOI:** 10.1038/s41419-021-03426-z

**Published:** 2021-02-04

**Authors:** Luisa Klemke, Tiago De Oliveira, Daria Witt, Nadine Winkler, Hanibal Bohnenberger, Richard Bucala, Lena-Christin Conradi, Ramona Schulz-Heddergott

**Affiliations:** 1 Institute of Molecular Oncology, University Medical Center Göttingen, Göttingen, Germany; 2 Department of General, Visceral, and Pediatric Surgery, University Medical Center Göttingen, Göttingen, Germany; 3 Institute of Pathology, University Medical Center Göttingen, Göttingen, Germany; 4 Departments of Medicine, Pathology, and Epidemiology & Public Health, Yale School of Medicine and Yale Cancer Center, New Haven, CT USA

**Keywords:** Colorectal cancer, Chemokines

## Abstract

Macrophage migration inhibitory factor (MIF) is an upstream regulator of innate immunity, but its expression is increased in some cancers via stabilization with HSP90-associated chaperones. Here, we show that MIF stabilization is tumor-specific in an acute colitis-associated colorectal cancer (CRC) mouse model, leading to tumor-specific functions and selective therapeutic vulnerabilities. Therefore, we demonstrate that a *Mif* deletion reduced CRC tumor growth. Further, we define a dual role for MIF in CRC tumor progression. *Mif* deletion protects mice from inflammation-associated tumor initiation, confirming the action of MIF on host inflammatory pathways; however, macrophage recruitment, neoangiogenesis, and proliferative responses are reduced in *Mif*-deficient tumors once the tumors are established. Thus, during neoplastic transformation, the function of MIF switches from a proinflammatory cytokine to an angiogenesis promoting factor within our experimental model. Mechanistically, *Mif*-containing tumor cells regulate angiogenic gene expression via a MIF/CD74/MAPK axis in vitro. Clinical correlation studies of CRC patients show the shortest overall survival for patients with high MIF levels in combination with CD74 expression. Pharmacological inhibition of HSP90 to reduce MIF levels decreased tumor growth in vivo, and selectively reduced the growth of organoids derived from murine and human tumors without affecting organoids derived from healthy epithelial cells. Therefore, novel, clinically relevant Hsp90 inhibitors provide therapeutic selectivity by interfering with tumorigenic MIF in tumor epithelial cells but not in normal cells. Furthermore, *Mif*-depleted colonic tumor organoids showed growth defects compared to wild-type organoids and were less susceptible toward HSP90 inhibitor treatment. Our data support that tumor-specific stabilization of MIF promotes CRC progression and allows MIF to become a potential and selective therapeutic target in CRC.

## Introduction

Macrophage migration inhibitory factor (MIF), which was originally discovered as a secreted proinflammatory cytokine with a central role in immune and inflammatory responses, has also been identified as a tumor promoter^1,2^. MIF is known to exert effects in epithelial cancer cells, stromal fibroblasts, endothelial cells, and immune cells^3–10^. In tumors, the major source of MIF is the epithelial cells themselves^11–13^, followed by a minor secretory contribution from constituents of the tumor microenvironment, such as stromal and inflammatory cells^5,14,15^. Therefore, tumor cells aberrantly elevate MIF expression via Hsp90-mediated protein stabilization^10,11,16^. The HSP90 chaperone machinery is a prerequisite for tumorigenesis because it stabilizes oncogenic and tumor-promoting proteins, protecting them from degradation^17,18^. We previously identified MIF as an Hsp90-stabilized protein in breast cancer cells^11^.

Colorectal cancer (CRC) patients also present elevated MIF levels, which are associated with a worse prognosis^12,15,19–22^. Among cancers, CRC has the third highest incidence^23^. Previous in vitro studies in human CRC cells showed that MIF increases proliferation, angiogenesis, and migration^12,24,25^. Functionally, MIF can bind to its main receptor CD74 to activate p38, MAPKs, or PI3K/AKT, which induces the expression of angiogenic factors^4,12,24,26–28^. Furthermore, MIF regulates therapeutic resistance via regulation of STAT3, MAPKs, AMPK, or hypoxia-dependent mechanisms^28–31^. Other studies using CT26 allograft models support that MIF promotes CRC progression^12,24^. In vivo, it has been shown that MIF stimulates the early stages of small intestinal adenomas in *Apc*^min^ mice^27^. Although all these studies showed a positive correlation between aberrant MIF function and CRC growth, an in vivo model of causative and severe CRC that mimics the human CRC was not available.

In our study, we investigated whether MIF promotes tumor growth in an autochthonous colorectal azoxymethane (AOM)/dextran sodium sulfate (DSS) mouse model and whether MIF can serve as a potential drug target. Because of the tumor-specific Hsp90-mediated stabilization of MIF, this protein could be selectively targeted in CRC. Our data suggest that MIF increases CRC growth and supports tumor-specific macrophage recruitment, tumor cell proliferation, and neoangiogenesis without affecting overall inflammation in established tumors.

Strikingly, a recent study in a mouse model of chronic colitis-dependent CRC reported a tumor-protective role for MIF^32^. This phenomenon was not observed in neither the *Apc*^min^ mouse model^27^ nor several other in vivo cancer studies, including myc-induced lymphoma, chronic lymphocytic leukemia, breast, prostate, bladder, and skin cancer^3,4,11,33–38^. An important difference between the previous work and our study is that we used a mouse model of acute colitis-associated CRC, which is more similar to human sporadic CRC^39^. Importantly, in our sporadic CRC model, MIF as a tumor-promoting factor is selectively targetable in tumor cells by inhibiting Hsp90, supporting a strong rationale for MIF as a potential therapeutic target in sporadic CRC.

## Results

### MIF supports tumor growth in a mouse model of CRC

**Fig. 1:**
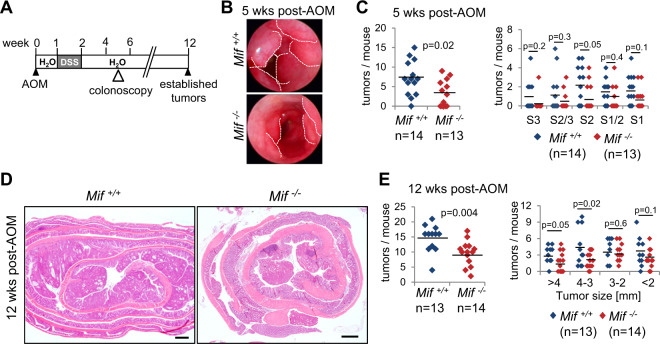
MIF supports tumor growth in a CRC mouse model. **A** Schematic of the AOM/DSS CRC mouse model. Visualization of tumor burden started at week 5 by colonoscopy. At 12 weeks post-AOM injection, established tumors were analyzed. **B** Representative colonoscopy images of the colonic lumen of the indicated genotypes at week 5 post-AOM. Dashed lines indicate the tumor borders. **C** Average number of tumors (left) and tumor sizes (right) in *Mif*^*+/+*^ and *Mif*^*−/−*^ mice at 5 weeks post-AOM injection. Numbers were determined by colonoscopy in living mice. S1 = small tumors to S3 = larger tumors. **D** Representative H&E-stained colonic tissues with tumors from *Mif*^*+/+*^ and *Mif*^*−/−*^ mice at week 12 post-AOM injection. Scale bars, 600 µm. **E** Average number of tumors per mouse (left) and tumor sizes (right) of the indicated mice at 12 weeks post-AOM injection. **C**, **E** *n*, mouse numbers. Black lines, mean. Student’s *t* test of indicated groups.

Given the importance of MIF in cancer and to determine whether MIF supports CRC tumorigenesis, we used the severe CRC AOM/DSS mouse model, which includes one phase of acute colitis (Fig. 1A). After a recovery phase, mice exclusively develop tumors within 12 weeks in the large intestine^40^. At 5 weeks post-AOM, when the tumors were macroscopically visualized by colonoscopy, *Mif*^*−/−*^ mice showed a reduction in the tumor burden (Fig. 1B). Quantification of colonic tumors by a scoring system^41,42^ revealed a reduction in tumor multiplicity in *Mif*^*−/−*^ mice (Fig. 1C). Moreover, at 12 weeks post-AOM, during which the CRC tumors are well established, MIF deficiency decreased tumor burden and numbers (Fig. 1D, E). In summary, MIF supports tumor growth in an acute colitis-associated CRC mouse model.

### MIF levels are elevated in CRC cells

**Fig. 2:**
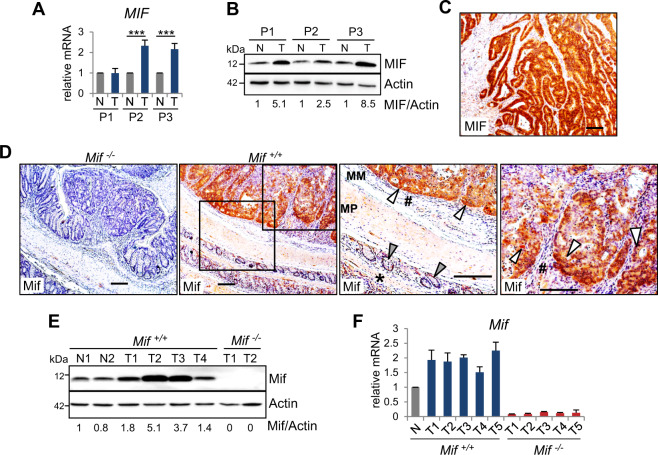
MIF levels are elevated in CRC cells. **A** Relative *MIF* mRNA expression of individual human CRC patients (P1–P3). Matched pairs of adjacent epithelia (‘N’) and their tumors (‘T’) were evaluated by qRT-PCR, and RNA levels were normalized to those of *RPLP0* mRNA. Mean ± SD of four technical replicates. Student’s *t* test was performed for comparison of indicated groups; ***p ≤ 0.001. **B** MIF protein levels in matched pairs of tumor (‘T’) and adjacent epithelial (‘N’) tissues from three individual human CRC patients (P1–P3). Immunoblot analysis. Actin, loading control. MIF expression ratios (MIF/Actin) were calculated by densitometry, normalized to the loading control, relative to the respective epithelium (‘N’). **C** Histological MIF staining in a colonic tumor from a human CRC patient. Scale bar, 200 µm. **D** Representative histological Mif staining in colonic tumors from *Mif*^*+/+*^ and *Mif*^*−/−*^ mice. *Mif*^*−/−*^ tumors served as negative control. Rectangles represent the area at a higher magnification. MM, muscularis mucosae; MP, muscularis propria. White arrows, tumor epithelial cells with elevated MIF levels. #, stromal cells of mucosae. Gray arrows, epithelial cells within the next surrounding colonic tissue layer. *, MM and MP of the next surrounding layer. Note that normal epithelial cells from untransformed regions of the colon show low MIF levels (gray arrow) compared to those in tumor cells (white arrow). Scale bars, 200 µm. **E** Mif protein levels in murine single tumor samples (‘T’) compared to matched normal epithelium (‘N’) from *Mif*^*+/+*^ mice. *Mif*^*−/−*^ tumors served as a negative staining control. Mif ratios were calculated as described in (**B**). **F** *Mif* mRNA level in single tumor samples (‘T’) of *Mif*^*+/+*^ and *Mif*^*−/−*^ mice compared to pooled normal epithelium (‘N’) of *Mif*^*+/+*^ mice (*n* = 2). mRNA levels were normalized to those of *Rplp0* mRNA. Means ± SD of four technical replicates.

During tumorigenesis, MIF protein levels are increased^12,27^. Our data confirm elevated MIF levels in cancer cells from CRC patients (Figs. 2A–C, S1A). Compared to the moderate increase in *MIF* mRNA levels (Figs. 2A, S1A), MIF protein levels were strongly increased in tumors from patients (Fig. 2B, C). Similar to the patient tumors, established AOM/DSS-induced tumors confirmed tumor-specific elevation of MIF expression (Fig. 2D). Intriguingly, epithelial cancer cells express high levels of MIF compared to those in the normal surrounding epithelium (Fig. 2D), indicating that the major source of MIF is tumor epithelial cells. Measurement of MIF expression in murine tumor lysates indicated increased MIF expression in tumors compared to normal colonic tissue (Figs. 2E, F and S1B, C). Taken together, these results confirm an enhanced tumor-specific increase in MIF occurrence within the epithelial tumor compartment.

### A *Mif* deletion protects mice from inflammation-associated cancer initiation

**Fig. 3:**
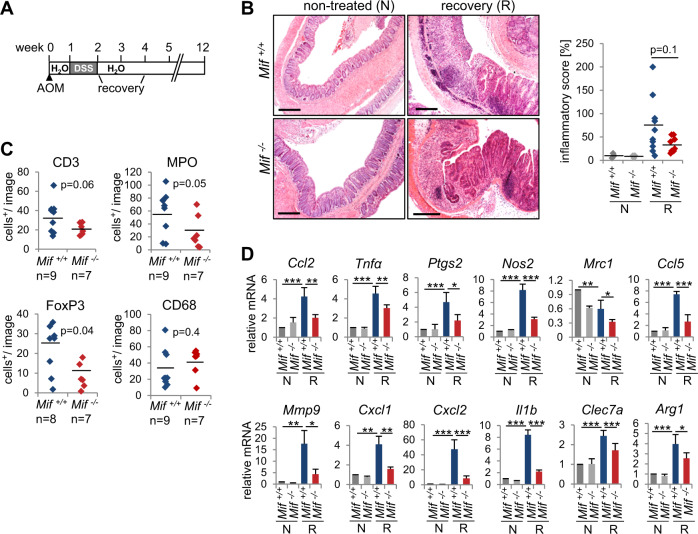
A *Mif* deletion protects mice from inflammation-associated cancer initiation. **A** Schematic of the AOM/DSS mouse model. CRC was initiated by a single AOM injection, followed by one cycle of DSS administration. At 8 days of post-DSS treatment, mice were analyzed for recovery. **B** Representative H&E staining of colonic tissues from the indicated mice 8 days post-DSS (recovery = ‘R’) or from untreated control mice (‘N’). Scale bars, 300 µm. (Right) The ‘inflammatory score’ was assessed based on tissue morphology and immune cell infiltration. (Left) Nontreated mice, *n* = 3/group; recovery *Mif*^*+/+*^ mice, *n* = 10, *Mif*^*−/−*^ mice, *n* = 8. Black lines, mean. *p* values were calculated with ANOVA Bonferroni’s multiple comparison test; *p* = 0.0455. Indicated groups, calculated with Student’s *t* test. **C** Quantification of the indicated histological staining of colonic tissues 8 days post-DSS (‘recovery’) in *Mif*^*+/+*^ and *Mif*^*−/−*^ mice. *n*, mouse numbers. Five to seven images (area = ×40 magnification) per mouse were analyzed for positively stained stromal cells. Black lines, mean. **D** mRNA levels of inflammatory genes in recovering (‘R’) and nontreated (‘N’) colonic tissues. Single samples from the indicated genotypes/groups were pooled (nontreated, *n* ≥ 3; recovering tissue, *n* ≥ 4). qRT-PCR was performed, and the expression levels were normalized to those of *Rplp0* mRNA. Means ± SD of ≥3 technical replicates/pools, each pipetted. **C**, **D** *p* values were calculated with Student’s *t* test comparing the indicated groups. *p ≤ 0.05, **p ≤ 0.01, ***p ≤ 0.001.

As a proinflammatory cytokine, MIF regulates immune responses and is suggested to be a link between inflammation and cancer^1,2^. Therefore, we hypothesized that the loss of *Mif* expression protects mice during the colitis-associated phase of tumor initiation. Indeed, during the recovery phase, colonic tissue damage and epithelial cell loss, as reflected by the inflammatory score, were increased in *Mif*^*+/+*^ mice compared to *Mif*^*−/*−^ mice and were accompanied by increased immune cell infiltration (Fig. 3A, B). To further examine the inflammatory response, we histologically analyzed the immune cell composition within the tumor microenvironment. Infiltrates from the colonic tissue of *Mif*^*+/+*^ mice had higher percentages of CD3-positive (T-lymphocyte marker), MPO-positive (neutrophil/granulocyte marker), and FoxP3-positive (regulatory T-cell marker) cells than did colonic tissue from *Mif*^*−/−*^ mice (Figs. 3C, S2A). Immune infiltrates and the inflammatory score showed a positive correlation (Figure S2B). Interestingly, CD68-positive (monocyte/macrophage marker) cell infiltration was unchanged between the two mice groups (Fig. 3C, S2A). Similar to the changes in the inflammatory cell composition, the expression of inflammation-associated cytokines was downregulated in *Mif*^*−/−*^ tissues during the recovery period, confirming a reduction in inflammation in the absence of Mif (Fig. 3D, S2C). Consistent with the protective effect of *Mif* deletion during recovery, *Mif*^*−/−*^ mice showed a reduced overall inflammatory response under DSS administration (Figures S2D-G). Furthermore, since MIF inhibits p53 activity^11,43^, we pursued whether MIF interferes with the DNA damage response and apoptosis in response to AOM treatment. Surprisingly, neither the levels of phosphorylated histone H2A.X (a DNA damage marker) nor the expression of p53 target genes (*Mdm2, Cdkn1a, Ccnd1, Gadd45a*, and *Bax*) was altered in colonic tissues in *Mif*^*−/−*^ mice compared to those in *Mif*^*+/+*^ mice, suggesting that MIF failed to regulate an AOM-induced p53-dependent response in colonic epithelia (Figures S2H, I).

Overall, a *Mif* deficiency protected mice during the early phases of inflammation in the AOM/DSS model and demonstrated that during colitis-associated tumor initiation, MIF acts as a proinflammatory cytokine.

### MIF supports CRC development via tumor-specific macrophage recruitment and angiogenesis without affecting overall inflammation

**Fig. 4:**
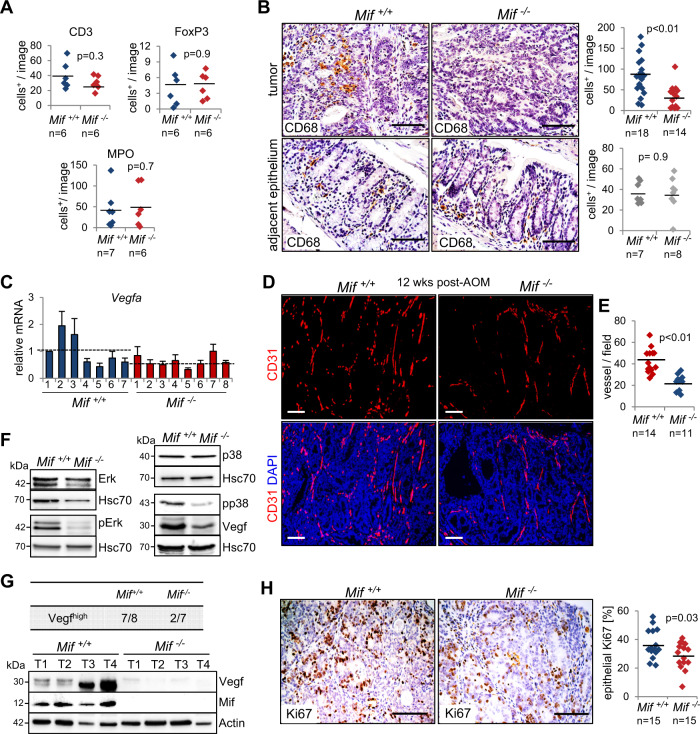
MIF accelerates proliferation and angiogenesis in established colorectal tumors. **A** Quantification of the indicated histological staining of *Mif*^*+/+*^ and *Mif*^*−/−*^ tumors at 12 weeks post-AOM treatment. At least 2 images (area = ×40 magnification) per tumor were viewed for positive stromal cells. *n*, tumor number from 3 mice each. **B** Representative CD68 staining of indicted tumors and their corresponding adjacent normal epithelium at 12 weeks post-AOM injection. Scale bars, 100 µm. (Right) For each tumor, ≥3 images were taken, and positive staining was quantified. There were 18 tumors from 7 *Mif*^*+/+*^ mice and 14 tumors from 8 *Mif*^*−/−*^ mice. For adjacent epithelium, ≥5 images (area = ×40 magnification) per mouse were viewed and counted for positive stromal cells. *n*, mouse numbers. **C** mRNA levels of *Vegfa* in single tumors of different mice (*Mif* ^+/+^, *n* = 7; *Mif*^*−/−*^ *n* = 8), relative to housekeeping gene, *Hprt1*. Means ± SD of four technical replicates. Black dashed line, mean. **D** Representative immunofluorescence for CD31 in tumors from *Mif*^*+/+*^ and *Mif*^*−/−*^ mice at 12 weeks post-AOM. DAPI, counterstaining. Scale bars, 100 µm. **E** Quantification of the vessel number from (**D**). Vessels from at least five images (area = ×40 magnification) per tumor were counted. A ‘vessel’ is defined as one separate fragment of CD31 staining. *n* = number of tumors out of 5 mice each. **F** Immunoblot analysis of *Mif*^*+/+*^ and *Mif*^*−/−*^ tumors at 12 weeks post-AOM with pooled samples (*n* ≥ 6 tumors per condition). Hsc70, loading control. **G** Summary of *Mif*^*+/+*^ (*n* = 8) and *Mif*^*−/−*^ (*n* = 7) tumors analyzed by immunoblot. (Bottom) Representative immunoblot analysis of single *Mif*^*+/+*^ and *Mif*^*−/−*^ tumors at 12 weeks post-AOM. Compared to a reference tumor (T1 of *Mif*^*+/+*^), ‘Vegf^high^’ means higher or same protein levels than the reference tumor. Actin, loading control. **H** Representative histological staining for Ki67 in tumors from *Mif*^*+/+*^ and *Mif*^*−/−*^ mice and quantification at 12 weeks post-AOM. Scale bars, 100 µm. Quantification of Ki67 staining counted within 2–4 images (area = ×40 magnification) per tumor. *n*, tumor number; both groups showed 15 tumors from 5 mice. **A**, **B**, **E**, **H** Black lines, mean. *p* values were calculated by Student’s *t* test.

To determine whether MIF also acts as an inflammatory cytokine to support established tumors, we analyzed the expression of inflammatory markers at 12 weeks post-AOM. Interestingly, immunohistological staining (Figure S3A) and their corresponding quantifications (Fig. 4A) did not show any differences in the extent of infiltrating lymphocytes, regulatory T-cells or neutrophils/granulocytes within established tumors. In line with these findings, an assessment of inflammatory cytokines from tumor lysates failed to show major differences between *Mif*-expressing and *Mif*-deficient tumors, although all cytokines were upregulated in tumor samples (‘T’) compared to normal epithelium samples from untreated animals (‘N’) (Figure S3B). However, interestingly, CD68-positive macrophage/monocyte infiltration was decreased in *Mif*^*−/−*^ tumors compared to *Mif*^*+/+*^ tumors (Fig. 4B, upper panel), supporting the function of MIF as a chemokine to mediate macrophage recruitment^3,4,44^. To clarify whether elevated MIF expression in tumor cells mediates tumor-specific macrophage recruitment, we monitored the adjacent epithelium (Fig. 4B, lower panel). Indeed, macrophages specifically infiltrated tumors, suggesting that MIF regulates the chemotaxis of tumor-associated macrophages to promote CRC tumorigenesis. Tumor-associated macrophages are known to secrete tumor-promoting cytokines during cancer progression to stimulate tumor cell proliferation and angiogenesis^45,46^. Indeed, levels of Vegfa, an angiogenic cytokine known to be secreted by macrophages^46,47^, are reduced in *Mif*^*−/−*^ tumors (Figs. 4C, F, G, S3C). Moreover, in established CRC tumors, *Mif*^*+/+*^ mice showed stronger vessel formation, as indicated by CD31-positive staining, compared to *Mif*^*−/−*^ mice (Fig. 4D, E). Immunoblots confirmed increased activation of proangiogenic factors such as p38 and ERK in Mif-containing samples (Fig. 4F), an effect described previously^3,10,12,27,48^. MIF also affected tumor cell proliferation in AOM/DSS-induced tumors (Fig. 4H), which might explain the smaller tumors observed in *Mif*^*−/−*^ mice (Fig. 1D, E).

Interestingly, Akt activity remained unchanged in *Mif*-deficient AOM/DSS tumors (Figure S3D), despite strong evidences that MIF activates PI3K/AKT in CRC^26^ and other cancers^49,50^.

Given that MIF also intrinsically regulates apoptosis via p53, e.g., in HER2-positive breast cancer or macrophages^11,51^, we clarified whether the loss of MIF expression also activates p53 target genes in AOM/DSS tumors. In our CRC mouse model, *Mif* deficiency did not upregulate the expression of p53 target genes involved in apoptosis (e.g., *Bax, Bcl2l1, Bcl2*, and *Mcl1*) (Figure S3E). TUNEL staining confirmed the lack of altered apoptosis in *Mif*^*−/−*^ tumors (Figure S3F). However, the expression of the cell cycle inhibitor *p21*/*Cdkn1a* was upregulated in *Mif*^*−/−*^ tumors (Figure S3E), supporting the diminished proliferation upon MIF loss (Fig. 4H).

To assess whether angiogenesis and proliferation are affected during the recovery phase, we evaluated *Vegfa*, CD31, and Ki67 expression in colonic tissues at 8 days post-DSS (Figures S3G-K) and found that neither vessel formation and *Vegfa* expression nor proliferation was dependent on the presence of MIF during colonic tissue recovery.

Albeit our data confirmed that MIF supports inflammatory processes during colitis-associated tumor-initiating phases, we identified that in established tumors, MIF contributes to tumor-specific macrophage recruitment, tumor cell proliferation, and vessel formation without affecting overall inflammatory responses. Whether these infiltrated macrophages release proangiogenic cytokines^45,47^ or whether MIF regulates angiogenic pathways in tumor cells themselves^52^ must be further elucidated.

### The CD74-MIF receptor complex facilitates the expression of proangiogenic factors in human CRC cells

**Fig. 5:**
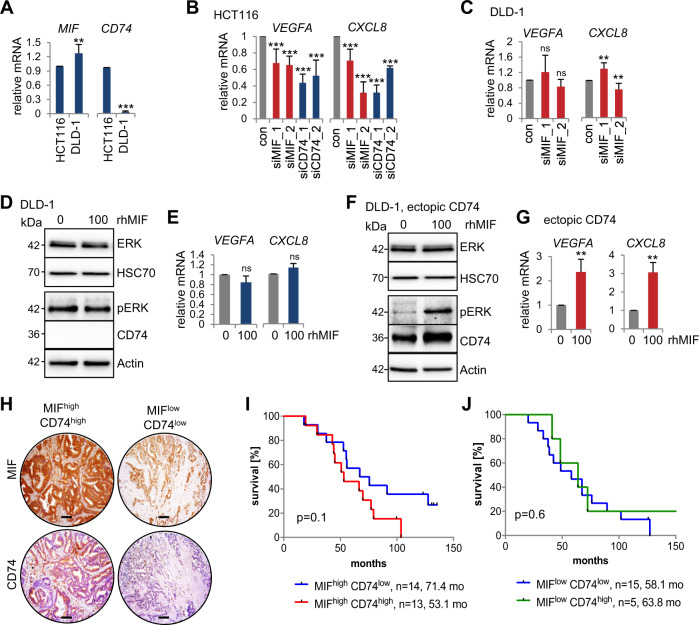
The CD74-MIF receptor interaction facilitates the expression of angiogenic factors in CRC cells. **A** qRT-PCR analysis of the relative mRNA levels of *MIF* and *CD74* in HCT116 and DLD-1 CRC cells. Normalized to those of *RPLP0*. Means ± SD of 2 biological replicates with 3 technical replicates each. **B**, **C** Expression analysis of the indicated angiogenic genes in HCT116 (**B**) or DLD-1 (**C**) cells after siRNA-mediated knockdown of MIF or CD74 for 72 h; a scrambled siRNA served as the control (‘con’). qRT-PCR were normalized to *HPRT1* or *RPLP0*, respectively. Means ± SD of ≥3 technical replicates from two biological replicates. **D**, **F** Angiogenic pathway analysis in CD74-deficient DLD-1 cells. 48 h after DLD-1 cells were transfected with an empty control (**D**) or CD74 overexpression plasmid (**F**), they were treated with 100 ng/mL recombinant human MIF (rhMIF) for 24 h. Immunoblots of CD74, pERK, and total ERK. Actin and Hsc70, loading controls. **E**, **G** Angiogenic marker expression (*VEGFA*, *CXCL8*). DLD-1 cells were treated as described in (**D**, **F**) followed by qRT-PCR. Expression of the indicated genes was normalized to that of *RPLP0*. Means ± SD of ≥3 technical replicates. **H** Representative images of serial sections from two human CRC patients stained for MIF and CD74 with different stabilization of MIF (MIF^high^: stabilized, MIF^low^: unstabilized) and expression levels of CD74 (CD74^high^, CD74^low^). Scale bars, 200 µm. **I**, **J** Correlation between patients with stabilized MIF (MIF^high^) and CD74 levels (CD74^high^, CD74^low^) (**I**) and patients with unstabilized MIF (MIF^low^) and CD74 levels (CD74^high^, CD74^low^) (**J**) on overall survival of patients. Log-rank (Mantel-Cox) test for comparison of indicated groups. **A**, **B**, **C**, **E**, **G** *p* values were calculated with Student’s *t* tests for comparison of indicated groups; ns = not significant, *p ≤ 0.05, **p ≤ 0.01, ***p ≤ 0.001.

MIF functions through CD74/CD44 and/or CXCR2/4 receptor complexes in proliferation, angiogenesis, and with its chemokine-like properties in monocyte and leukocyte recruitment^3,8,53–55^. The CD74 receptor is the main MIF receptor^53,56^. Since the expression of *Vegfa* is downregulated in *Mif*-deficient tumors (Figs. 4C, S3C), we examined whether tumor cells themselves are able to express angiogenic genes via MIF binding to CD74 to activate MAP kinases to induce *VEGF* and *IL8* expression^12,24,26–28^. First, we used the CD74-expressing (Fig. 5A) and MIF secreting HCT116 cell line^29,57^. Indeed, knockdown of either MIF or CD74 in HCT116 cells reduced *VEGFA* and *CXCL8/IL8* expression supporting a MIF-CD74 axis (Figs. S4A, 5B). Second, we used DLD-1 cells which do not express CD74 and are not shown to secrete MIF (Fig. 5A), thus, missing the prerequisites (secreted MIF and CD74) for a MIF-CD74 axis. As expected, in parental DLD-1 cells, depletion of MIF did not show any alterations in *VEGFA* and *CXCL8/IL8* expression (Figs. S4B, 5C). Moreover, supplementation of recombinant MIF (rhMIF) in DLD-1 cells to mimic MIF secretion, also failed to activate ERK or angiogenic gene expression (Fig. 5D, E). Importantly, supplementation of both, MIF by rhMIF and CD74 by plasmid-based ectopic expression, lead to ERK activation and increased *VEGFA* and *CXCL8/IL8* expression confirming that concomitant CD74 and secreted MIF are necessary for expression of angiogenic markers (Fig. 5F, G). To further investigate the MIF-CD74 axis, we performed clinical correlation studies based on MIF and CD74 expression levels of human CRC patients (Fig. 5H, I, J). Interestingly, simultaneous high levels of MIF and CD74 showed a trend for patient shortest survival (53.1 months) compared to stabilized MIF alone (71.4 months) (Fig. 5I). In contrast, CD74 status in patients with low MIF levels did not impact overall survival (Fig. 5J).

These findings underline the importance of MIF in cancer and support that MIF acts via CD74 in CRC.

### MIF-driven CRC is vulnerable to Hsp90 inhibition

**Fig. 6:**
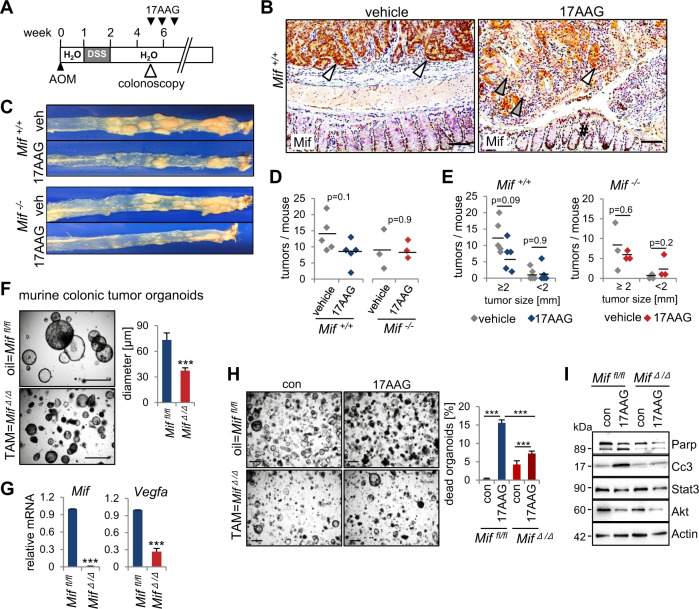
MIF-driven CRC is vulnerable to Hsp90 inhibition. **A** Hsp90 inhibitor treatment timeline within the AOM/DSS mouse model. After tumors visualized via colonoscopy and scored as more than one S2/3 or three S1/2 tumors, mice were treated with the HSP90 inhibitor 17AAG for 3 weeks. **B** Representative histological Mif staining of tumor-bearing colonic tissue from *Mif*^*+/+*^ mice after 3 weeks of 17AAG or vehicle treatment. White arrows indicate tumor epithelial cells with elevated Mif levels. Gray arrows indicate tumor cells with degraded Mif. #, normal epithelia of the next surrounding layer with low Mif levels. Scale bars, 100 µm. **C** Macroscopic overview of entire tumor-bearing colons from *Mif*^*+/+*^ and *Mif*^*−/−*^ mice after 3 weeks of treatment with 17AAG or vehicle. **D** Numbers of tumors per mouse as indicated in *Mif*^*+/+*^ (*n* = 5 mice per group) and *Mif*^*−/−*^ mice (*n* = 3 per group). Black lines, mean. *p* values with ANOVA, Bonferroni’s multiple comparison test, *p* = 0.212. **E** Tumor sizes of mice from (D). Indicated groups, calculated with Student’s *t* test. **F** Assessment of organoid growth in *Mif*-depleted organoids. *Mif*^*fl/fl*^*;villincreERT2* mice with a defined tumor burden were treated with Tamoxifen (TAM) or respective vehicle control to generate *Mif*-deficient CRC tumors (*Mif*^*∆/∆*^) to mimic a *Mif* epithelial-specific knockout. From those tumors, colonic tumor organoids were generated. Representative images and quantification of colonic tumor organoids (p 5) derived from oil (*Mif*^*fl/fl*^) or TAM (*Mif*^*∆/∆*^) treated mice. (right) At least 11 images were quantified from 10 different gel domes. Diameter of organoids was measured using ImageJ. Scale bars, 200 µm. Means ± SD from different images. **G** Expression analysis of *Mif* and *Vegfa* in *Mif*^*fl/fl*^ or *Mif*^*∆/∆*^ derived organoids. Via qRT-PCR, expression was co-normalized to *Rplp0* and *Hprt1*. Means ± SD of 4–5 technical replicates. **H** Imaging and quantification of organoid death of *Mif*-proficient (top) and *Mif*-deficient (bottom) organoids prepared as in (**F**) after 17AAG (500 nM) for 21 h. Scale bars, 200 µm. Quantifications based on organoid morphology. At least 5 images from ≥5 gel domes (×4 magnification each) per group were counted. The percentage of dead organoids was calculated to all organoids. Mean ± SD from ≥6 images. **I** Immunoblot of organoids of (**H**) at the endpoint of the experiment. Cc3 (cleaved caspase-3) and Parp (poly(ADP-ribose)-polymerase), apoptotic markers. Stat3 and Akt, further Hsp90 clients served as positive control for 17AAG functionality. Actin, loading control. **F**, **G**, **H** *p* values, *Student*’*s* t tests for indicated groups.

Next, we asked whether constitutive MIF stabilization in CRC cells creates vulnerabilities that can be therapeutically targeted. Since MIF is stabilized by Hsp90^11^, we used the pharmacological Hsp90 inhibitor 17AAG. When *Mif*^*+/+*^ and *Mif*^−*/*−^ mice reached a defined tumor burden, they were treated with 17AAG (Fig. 6A). Hsp90 inhibition reduced MIF protein levels in AOM/DSS tumors (Fig. 6B) and showed a trend for decreased tumor burden in *Mif*^*+/+*^ mice (Fig. 6C–E). Differences were not statistically significant but showed a trend in *Mif*^*+/+*^ mice (Fig. 6D, E, left panels). By contrast, Hsp90 inhibition in *Mif*^*−/−*^ mice failed to achieve tumor reduction (Fig. 6D, E, right panels).

To further support MIF as tumor-relevant Hsp90 client in CRC progression, we used genetically deleted MIF tumor organoid cultures. We observed a decreased growth in *Mif*-depleted organoids (Fig. 6F, G), further confirming, that MIF loss reduces tumor cell proliferation (Fig. 4H). Whether these growth defects arise from intracellular MIF functions and/or an MIF-CD74 axis remains elusive. Moreover, *Vegfa* expression was reduced in those organoids (Fig. 6G). To further support MIF as a tumor-relevant Hsp90 substrate in CRC, we analyzed these *Mif*-depleted organoids after treatment with 17AAG (Fig. 6G, H). Indeed, a *Mif* depletion led to a decreased susceptibility toward 17AAG treatment compared to *Mif*-proficient organoids (Fig. 6H). Furthermore, apoptotic markers such as cleaved caspase-3 and Parp were only upregulated after 17AAG treatment in *Mif*-proficient organoids, but not in *Mif*-deficient organoids (Fig. 6I).

These data support a relevant point: Hsp90 inhibition seems to stronger target CRC tumors with elevated MIF, although the HSP90 system stabilizes innumerable oncogenes. These findings support that MIF is a tumor-relevant Hsp90 substrate in CRC.

### MIF is a selective therapeutic target of Hsp90 inhibition in CRC-derived organoids

**Fig. 7:**
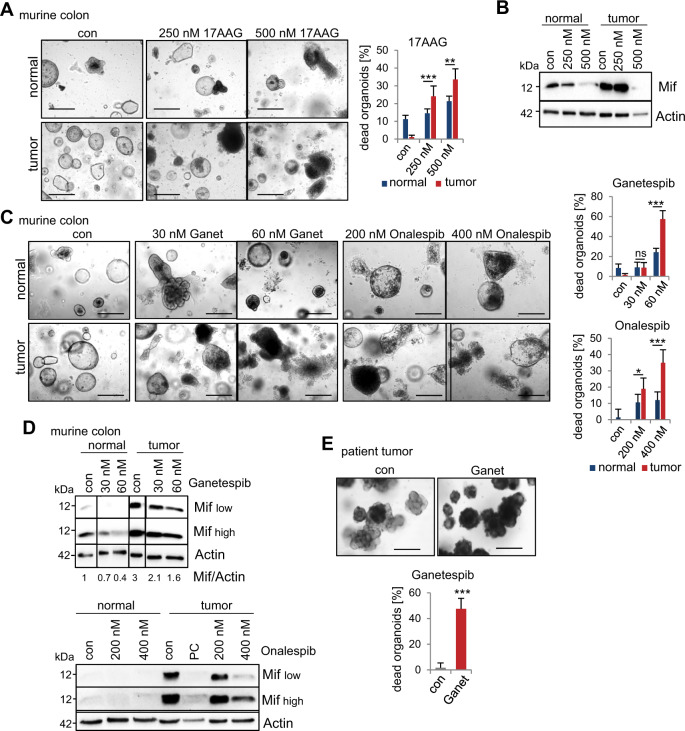
MIF is an actionable and selective therapeutic target via Hsp90 inhibition in CRC-derived organoids. **A**, **C** Therapeutic selectivity of Hsp90 inhibitors. Representative images show colonic organoids derived from matched pairs of pooled *Mif*^*+/+*^ tumors or their adjacent epithelium (‘normal’). The indicated organoids were treated with DMSO (‘con’), 17AAG (**A**), Ganetespib (‘Ganet’), or Onalespib (**C**) at the indicated concentrations for 21 h. Scale bars, 200 µm. (Right) Quantifications based on organoid morphology. At least 6 images from ≥5 gel domes (×4 magnification each) per group were counted. For each group, number of dead organoids was calculated by the number of total organoids. The data are presented as the means ± SD from different images. **B**, **D** Hsp90 inhibitor treatment as described in (**A**, **C**) of matched pairs. Immunoblot analysis was performed to evaluate Mif degradation. ‘Mif high’ and ‘Mif low’ indicate exposure times during signal acquisition. Actin, loading control. PC, positive control. Mif/Actin, Mif expression ratios compared to Actin. **E** Representative images and quantification of organoids from patients with resectable CRC treated with 80 nM Ganetespib for 21 h. Scale bars, 200 µm. Quantification was performed as described in (**A**). Five images (×10 magnification) per condition were quantified. Means ± SD from different images. **A**, **C**, **E** Student’s *t* test was performed for comparison of indicated groups; *p ≤ 0.05, **p ≤ 0.01, ***p ≤ 0.001.

To exploit further therapeutically targeting of stabilized MIF, we administered Hsp90 inhibitors to healthy epithelial/mucosal-derived and tumor-derived murine colonic organoids from the same AOM/DSS-induced mice (i.e., matched pairs). Since organoids derived from mice with a 129S1/SvImJ background failed to grow in vitro in our laboratory (Figure S5A), we used C57BL/6 mice. Observation of the organoid morphology and the subsequent quantifications showed higher levels of cell death after 17AAG in tumor-derived organoids, compared to the epithelial-derived organoids (Fig. 7A). Immunoblot analysis confirmed strong reduction of Mif levels especially after treatment with 500 nM 17AAG (Fig. 7B). This prompted us to test Ganetespib and Onalespib, two clinically relevant second-generation HSP90 inhibitors that have been extensively tested in clinical trials and have a suitable toxicity profile^58–61^. Both inhibitors induced cell death to a far lesser extent in normal epithelial-derived organoids than in tumor-derived organoids (Fig. 7C) and showed promising specificity toward tumor organoids. Although Mif protein was degraded by Hsp90 inhibition in normal and tumor-derived organoids treated with either inhibitor (Fig. 7D), only tumor-derived organoids were morphologically disrupted upon Hsp90 inhibition (Fig. 7C), indicating that MIF plays a tumorigenic role rather than an essential function in normal epithelial cells. Importantly, and in line with our findings, we confirmed the enhanced Mif levels in tumor-derived organoids (Fig. 7B, D). Furthermore, in MIF-expressing patient-derived CRC organoids, Ganetespib markedly increased organoid death compared to that observed in the control organoids (Fig. 7E).

Therapeutic selectivity toward tumor cells plays an important role in therapy implementation. To further test whether Hsp90 inhibitors affect healthy tissues, we used organoids derived from the murine small intestine. Upon implementing the same treatment scheme as that used for colonic tumor-derived organoids, we discovered that 17AAG, Ganetespib, and Onalespib only exerted minor or no effects on the small intestine-derived organoids (Figure S5B). Indeed, Ganetespib failed to significantly degrade Mif protein in those organoids (Figure S5C), while another Hsp90 substrate, Stat3, was degraded. This confirms the selectivity of Hsp90 inhibition toward stabilized MIF in tumors. Even though immuno blot analysis also showed reduction of Mif levels after 17AAG treatment (Figure S5C), again depletion of unstabilized Mif in small intestinal organoids, did not impact morphology or survival of organoids (Figure S5B) as observed for normal colonic epithelia-derived organoids (Fig. 7A, B).

Thus, our findings highlight that MIF degradation via Hsp90 inhibition is a promising mechanism in CRC therapy. MIF acts not only as a critical driver in CRC but also as a selective target for Hsp90 inhibition in tumors.

## Discussion

We used the immune-competent AOM/DSS mouse model, which mimics CRC progression in humans, to exploit the therapeutic potential of MIF. We demonstrated that MIF is specifically elevated in tumor cells and drives tumor growth in this acute colitis-associated (‘sporadic’) CRC model. Thus, in established tumors, stabilized MIF preferentially supports tumor-specific macrophage infiltration, vessel formation, and tumor cell proliferation.

Concomitantly, we also showed within this model that MIF regulates overall inflammatory signatures but especially during tumor initiation. Compared with *Mif*^*+/+*^ mice, *Mif*^*−/−*^ mice were protected against acute colitis-associated tumor initiation (Fig. 3), confirming the general function of MIF as a proinflammatory cytokine^3,10^. By contrast, established *Mif*-deficient tumors did not show reductions in overall inflammation (Fig. 4); rather, only tumor-associated macrophages significantly infiltrated *Mif*^*+/+*^ tumors. Thus, MIF seems to lose its overall proinflammatory function once CRC tumors are established. Proliferation, vessel formation and angiogenic cytokine expression were reduced in *Mif*-deficient tumors, an effect described previously^3,10,27,48^.

Studies showing that human tumor cells themselves are able to activate MAPK-mediated *IL8* and *VEGF* expression by binding of MIF to its main receptor CD74^7,12,27,28,35,53^ were confirmed within this study in human CRC cells (Fig. 5). Our data support that MIF can act in an autocrine MIF-CD74 manner in HCT116 CRC cells, resulting in accelerated expression of angiogenic factors. Furthermore, in DLD-1 cells, we supplemented recombinant MIF concomitantly with ectopic CD74 which mimics paracrine MIF-CD74 interactions to induce *VEGF* and *IL8*. In the in vivo CRC model, we assume that tumor epithelial cells do both, secrete MIF to recruit macrophages to the tumor (which consequently secrete angiogenic factors) (Fig. 4B); and provide an autocrine MIF-CD74 interaction to induce the MAPK-VEGF axis (Fig. 5B and F), albeit we have not specifically tested it in this study. However, reduced expression of VEGF in *Mif*-deficient organoids (Fig. 6G), support the idea, that tumor cells themselves contribute to VEGF expression. Nevertheless, MIF is known to act as chemokine on tumor-specific macrophage recruitment and/or macrophage polarization, and macrophages are known to secrete angiogenic factors, further promoting CRC tumorigenesis^44,55^. In sum, tumor cells and tumor-associated macrophages might contribute to angiogenic factor expression but stabilized MIF in epithelial tumor cells provides the prerequisite for both scenarios.

To further test whether tumor epithelial cells with elevated MIF expression provide dual control over tumor growth, additional experimental models with inducible, tissue-specific *Mif* deletions are required. In principle, reduced chemotaxis of *Mif*^*−/−*^ macrophages^62^ or *Mif*-depleted fibroblasts within the tumor stroma^63^ might also contribute to tumor reduction in *Mif*^*−/−*^ mice.

The co-expression of MIF and CD74 seems to be important in tumorigenesis (Fig. 5), and either MIF or CD74 alone might not be strong tumor biomarkers. Our CRC patient study (Fig. 5H-J) as well as patient studies of lung cancer and colon carcinomatosis indicate that MIF/CD74 co-expression corresponds to an even worse prognosis^28,64^. Moreover, a recent mouse study revealed a strong upregulation of CD74 during colonic inflammation, promoting mucosal healing, and epithelial tissue recovery by enhanced cell proliferation^65^. While this study confirms the importance of a MIF/CD74 co-existence in proliferation, it also clarified that a CD74 deficiency alone massively increases overall inflammation with a reduced recovery rate^65^. In contrast, MIF deletion or ablation alone protects against inflammation, demonstrated in experimental models of gastrointestinal inflammation^66–68^. Why a CD74 single deletion intensifies inflammation remains speculative^65,69^. One explanation might be altered macrophage recruitment. MIF^−/−^ macrophages exhibited reduced overall chemotaxis compared to wild-type macrophages, whereas CD74^−/−^ macrophages showed random chemokinesis^62^, leading to an accelerated inflammatory response. Moreover, receptors often co-regulate each other, and after CD74 loss, MIF might increase its affinity to CXCR2 and/or CXCR4 receptors driving inflammation instead of proliferation and angiogenesis^54,70^. Dual roles for ligand-receptor complexes are becoming increasingly evident in the context of active inflammation and mucosal recovery^69^. In sum, MIF/CD74 co-expression might be the major predictor for tumor growth in CRC.

MIF is mainly stabilized in tumors but not stromal or inflammatory cells (Fig. 2). MIF stabilization occurs via binding to Hsp90^16^, which offers therapeutic approaches to target cancer cells via Hsp90 inhibition. We showed for the first time that clinically relevant Hsp90 inhibitors decreased MIF levels in CRC and subsequently reduced tumor growth (Figs. 6 and 7). Given the plethora of known Hsp90-stabilized oncogenes^18^, it is interesting to see that Hsp90-mediated stabilization of MIF is critical for the survival of *Mif*-proficient murine colonic tumor-derived organoids (Fig. 6H). MIF reduced tumor-derived organoids show a reduced antitumor response to Hsp90 interference compared to that in *Mif*-proficient organoids, indicating that MIF is an important Hsp90-stabilized protein in CRC. Moreover, Hsp90 interference provides therapeutic selectivity toward tumor cells (Fig. 7). Since Hsp90 inhibitors exhibit fundamental differences in action^71^, we focused on newly developed inhibitors such as Ganetespib and Onalespib.

In summary, since MIF stabilization is a crucial event, specifically in tumor cells, Hsp90 inhibition provides a potential approach to target MIF function in CRC. These findings support the tumor-promoting role of MIF in CRC and highlight the necessity to better understand the underlying MIF-induced tumorigenic mechanisms in CRC.

## Materials and methods

### Patient samples

Clinical samples (protein samples, RNA samples, PFA-fixed paraffin-embedded sections, patient tissue for cultivation) were provided by the Department of General, Visceral and Pediatric Surgery of the University Medical Center Göttingen (UMG, Germany).

### Mouse models and genotyping

Mouse experiments were approved by state (Niedersächsisches Landesamt für Verbraucherschutz und Lebensmittelsicherheit, LAVES, Germany) and institutional (Göttingen University Medical Center) committees, which ensured that all experiments conformed to the relevant regulatory standards. Constitutive Mif knockout in the 129S1/SvImJ background has been described in detail in ref. ^72^. DNA isolation and genotyping were performed using DirectPCR lysis Reagent and OneTaq®Quick-Load® MasterMix. Genotyping primers are specified in Supplemental Table 1. *Mif*^*flox/flox*^ mice in C57BL/6NCrl background were described in detail^72,73^; and were used for the development of murine organoids. In brief, to remove floxed MIF alleles from colonic epithelial tissue, we crossed *Mif*^*fl/fl*^ mice with *villinCreERT2*-harboring mice to generate *Mif*^*fl/fl*^*;villincreERT2* transgenic mice. Mice were housed and handled under pathogen-free barrier conditions.

### Murine CRC induction, colonoscopy, and treatment

For experiments, randomly selected 10-week-old female and male mice (>20 g) were used. For the induction of colorectal tumors, mice were treated with a single intraperitoneal injection of 10 mg/kg azoxymethane (AOM, Sigma-Aldrich) in 0.9% sodium chloride. After 1 week of rest, 1.5% (129S1/SvImJ background) or 2% (C57BL/6 background) DSS (MP-Biomedicals) was added to the drinking water for 6 consecutive days. Throughout the AOM/DSS phase, the body weights of the mice were continuously measured.

Five weeks after AOM induction, tumor development was monitored weekly by conducting a colonoscopy (Karl Storz GmbH) on anesthetized mice (1.5–2% isoflurane inhalation). Tumor sizes were determined according to the method described by Becker and Neurath^41^ based on the colonic luminal perimeter as follows: S1 = just detectable, S2 = 1/8 of the lumen, S3 = 1/4 of the lumen, S4 = 1/2 of the lumen, and S5 > 1/2 of the lumen. For analysis of established tumors, we chose an endpoint study design, terminating the experiment at 12 weeks after AOM treatment to avoid losing mice to extraneous reasons such as intestinal obstruction and anal prolapse.

For pharmacological Hsp90 inhibitor analysis, tumors were visualized and validated by colonoscopy. Reaching a defined tumor burden, at least one S2/3 tumor and at least three S1/2 tumors when scored by colonoscopy, mice were treated with 17-allylamino, 17-demethoxygeldanamycin (17AAG, provided by the National Cancer Institute, NCI). Therefore, 17AAG was pre-dissolved in DMSO and further diluted in 10% DMSO/18% Kolliphor® RH40/3.6% Dextrose (Sigma-Aldrich) in H_2_O. 60 mg/kg of 17AAG or vehicle were given by intraperitoneal injection for 5 days per week for 3 consecutive weeks. During 17AAG treatment, tumors were weekly visualized and monitored by colonoscopy.

At endpoints, mice were euthanized and the entire large intestine was harvested, longitudinally opened, and displayed. Tumor numbers were counted and sizes were measured with an electronic caliper. For subsequent analysis, single tumor biopsies were taken. Each large intestine was ‘swiss rolled’, fixed in 3.7% paraformaldehyde/PBS, processed for embedding and bisected. Both halves were placed into a mold for paraffin embedding.

For *Mif* depletion in vivo, AOM/DSS-treated *Mif*^*fl/fl*^ mice were used for Tamoxifen (TAM, Sigma-Aldrich) or respective vehicle control (oil). Reaching a defined tumor burden, at least one S2/3 tumor and at least three S1/2 tumors when scored by colonoscopy, mice were treated for 5 consecutive days, followed by 2 days of rest and another 3 days TAM/oil treatment. Twelve days after TAM-end, mice were dissected, and organoids were prepared (see section above).

All animal experiments were carried out in full agreement with the guidelines outlined above.

### Human cell cultures, treatment, and transfection

The human CRC cell line DLD-1 was cultured in RPMI 1640 medium, whereas HCT116 CRC cells were cultured in McCoy’s 5A modified medium. Media were supplemented with 10% FBS, Penicillin-Streptomycin, and L-glutamine (RPMI 1640). Cell lines were cultured at 37 °C and 5% CO_2_ in a humidified atmosphere and were regularly tested for *Mycoplasma* contamination.

Knockdown of MIF or CD74 was achieved by siRNA transfection using Lipofectamine™3000 reagent according to the manufacturer’s instructions. All siRNAs were purchased from Ambion and used according their guidelines; the sequences are listed in Supplemental Table 2. CD74 overexpression in DLD-1 cells was performed using Lipofectamine™3000 transfection reagent. In brief, 24 h after cell seeding, DLD-1 cells were cotransfected with 0.5 μg of GFP-containing plasmid and 1.5 μg of either pcDNA3.1-CD74 expression plasmid^56^ or the corresponding pcDNA3.1/V5-His-TOPO control plasmid. Forty-eight hours post-transfection, cells were treated with recombinant human MIF as indicated.

### HEK293T cell media conditioning for organoid culture medium

HEK293T cells expressing murine R-spondin-1 and Noggin or Wnt3a were cultivated in DMEM supplemented with 10% FBS, Penicillin-Streptomycin and Sodium Pyruvate in a humidified atmosphere at 37 °C with 5% CO_2_. For HEK293T-mR-spondin-1 Zeocin and for HEK293T-mNoggin Geneticin were added to the medium during cultivation and expansion. For conditioning, medium was replaced by advanced DMEM/F12 medium supplemented with GlutaMAX™, Penicillin-Streptomycin, and 10 mM HEPES, and cells were cultivated for 1 week. For murine R-spondin-1-containing and Noggin-containing media, batch quality was examined using Dot-blot analysis. Murine colonic organoid culture medium contains advanced DMEM/F12 medium supplemented with 50% conditioned Wnt3a medium, 20% conditioned Noggin medium, 10% conditioned R-spondin-1 medium, N-2, B-27, 3.4 μg/mL ROCK inhibitor, 5 μM CHIR 99021, 500 nM A83-01, 10 mM Nicotinamide, 80 µM N-Acetyl-L-Cysteine, and 200 ng/mL rmEGF.

### Preparation and cultivation of colonic and small intestinal organoids

Tumor-harboring large intestines of C57BL/6 mice were harvested. Three to four tumors per mouse and in parallel, parts of the normal epithelium were biopsied from the same mouse allowing generation of matched organoid pairs. Normal epithelial tissue was cut, washed, and incubated in 4 mM EDTA/PBS for 30 min on ice. Pieces were thoroughly, mechanically dissociated in PBS. Tumor samples were digested with 2 mg/mL Collagenase type-I solution at 37 °C for 30 min. Normal crypts and tumor fragments were filtered using cell strainers. Cell pellets were washed, resuspended in cold Matrigel, and droplet-plated allowing Matrigel polymerization at 37 °C for 30 min. Organoids-containing domes were covered with organoid culture medium, cultivated at humidified 37 °C with 5% CO_2_. Medium was exchanged every 2 to 3 days. Organoids splitting was performed once a week. For passaging, organoids were manually disrupted in ice-cold PBS, and cultured as described above.

Small intestinal tract starting from jejunum to the end of ileum were prepared from C57BL/6 mice, incubated in 5 mM EDTA/PBS for 30 min on ice, washed, and mechanically dissociated. Crypts were resuspended in cold Matrigel and cultured as colonic organoids, but with small intestinal organoid medium containing advanced DMEM/F12 supplemented with 20% conditioned mNoggin medium, 10% conditioned R-spondin-1 medium, N-2, B-27, 80 µM N-Acetyl-L-Cysteine, 50 ng/mL rmEGF.

### Organoid treatments and morphological quantification

Experiment with matched pairs of normal epithelia-derived and tumor-derived colonic organoids, murine small intestinal organoids, and human organoids were performed between passage 2–7. HSP90 inhibitors 17AAG (National Cancer Institute, NCI), Onalespib and Ganetespib (Synta Pharmaceuticals) were dissolved in DMSO and used as indicated. For quantification of treatment response, light microscopy images of ≥5 Matrigel domes were taken from each condition. The amount of images was dependent on size and culture density as indicated. Based on morphology, dead organoids were defined as organoids with a partial or complete loss of outer epithelial barrier leading to disruption into clumps of dead cells or separation of dead cells^74^. The percentage of dead organoids was calculated relative to the total amount of organoids per image. For dead organoid quantification and measurement of organoid diameter ImageJ was used. For analysis of organoid lysates, Matrigel domes were disrupted using ice-cold PBS. Suspension was centrifuged and organoid-containing pellets were further washed and incubated with Cell Recovery solution (Corning) for complete removal of Matrigel. Organoids were resuspended in standard RIPA buffer for protein lysates and in TRIZOL for RNA isolation.

### Histological analysis

Immunohistological stainings were performed with standardized protocols for formalin-fixed paraffin-embedded (FFPE) tissues. Following primary antibodies were used: MIF (Sigma-Aldrich, HPA003868), CD74 (Sigma-Aldrich, HPA010592), phospho-histone H2A.X (Ser139, Cell Signaling, #9718), Ki67 (Abcam, ab15580), Cluster of differentiation 31 (CD31 (SZ31), Dianova, DIA-310), Cluster of differentiation 3 (CD3 [SP7], Abcam, ab16669), Forkhead-box protein p3 (FoxP3, Abcam, ab54501), Myeloperoxidase (MPO, R&D Systems, AF3667). For CD68, two different antibodies were used to double-check staining (Abcam, ab53444 and eBioscience™, 14-0681-82). For detection of primary antibodies from rabbit and rat, ImmPRESS™ Reagent anti-Rabbit IgG and ImmPressTM Reagent anti-Rat IgG (both Vector Laboratories) were used. For antibodies from goat, the ABC detection system was used, entailing a biotinylated goat/sheep antibody (GE Healthcare) and ExtrAvidin®−Peroxidase (Sigma-Aldrich). As substrate for the horseradish peroxidases served 3,3′-Diaminobenzidine tetrahydrochloride (DAB, Roth). Counterstain of the nuclei was achieved using Mayers Hämalaun solution (Merck). Alexa Fluor®594-coupled secondary antibody was used as detection system for immunofluorescence with DAPI (Sigma-Aldrich) as counterstain for nuclei. Images were taken using a standard microscope (Carl Zeiss AG) with the ZEN imaging program from Zeiss. Figures were further prepared using Adobe Photoshop software. For quantification of staining, samples were blinded and positively stained cells were counted manually using CellCounter function of ImageJ. Percentage of epithelial Ki67-positive cells was determined relative to the total number of epithelial cells. For staining of CD31, CD68, CD3, FoxP3, and MPO, the number of positive cells was counted per image.

Hämalaun & Eosin G stained sections were used to determine the inflammatory score. The inflammatory score is based on morphological changes (grade of damage) of the tissue due to immune cell infiltration and epithelial layer disruption. Grade 0 = factor 0, no infiltration of immune cells, normal distribution of epithelia and amount of goblet cells; grade 1 = factor 1, minor infiltration of immune cells, epithelia is still intact and minor changes in goblet cell number; grade 2 = factor 2, moderate infiltration of inflammatory cells, epithelia is partly damaged and reduced number of goblet cells; grade 3 = factor 3, massive infiltration of immune cells, complete disruption/loss of epithelia and loss of goblet cells. For calculation, amount of tissue in percentage with respective grade of tissue damage was multiplied with the corresponding factor (factor 0–3). The obtained percentages were summed, resulting in a value for the inflammatory score (minimum 0–maximum 300) for each mouse. To ensure unbiased quantification, the inflammatory score was individually determined by one scientist and one pathologist.

TUNEL staining was used to detect DNA-strand breaks occurring during apoptotic cell death in established tumors. TUNEL reaction mix (Sigma-Aldrich) consists of TUNEL enzyme solution and TUNEL label mix. The assay was performed according to manufactures guidelines. DAPI served as counterstaining, slides were mounted with Fluorescent Mounting Medium (DakoCytomation).

### Quantitative immunohistochemistry on colon cancer patient samples

Section of a tissue micro array (TMA) for primary colonic tumors was kindly provided by the Department of Pathology of the University Medical Center Göttingen (UMG, Germany). According to described standard protocols for immunohistochemistry (see above), TMAs were stained for MIF (Sigma-Aldrich, HPA003868) and CD74 (Sigma-Aldrich, HPA010592). For CD74 staining tumors with more than 10% strongly positive stained cells or more than 40% overall stained cells with lower intensity were graded as high (CD74^high^). For MIF staining, biopsis with high intensity in more than 70% of cells were graded as MIF^high^. Biopsis with moderate or low intensity were graded as MIF^low^.

### Immunoblot analysis

For Whole lysates from human CRC cell lines and murine organoids were made with standard RIPA buffer (1% sodium deoxycholate, 10 mM EDTA, 1% Triton X-100, 0.1% SDS, 150 mM NaCl, 20 mM Tris-HCl pH 7.5, cOmplete^TM^ mini protease inhibitor cocktail and phosphatase inhibitor mix consisting of 2 mM Imidazol, 1 mM sodium orthovanadate, and 1 mM sodium fluoride) was used. For protein extraction from human and murine samples, tissues were minced, lysed in RIPA buffer, and further processed by sonication. For determination of protein concentrations using BCA protein assay (Pierce), samples were centrifuged and supernatants were collected. Equal protein concentrations were separated by SDS gel electrophoresis and transferred onto nitrocellulose membranes (Amersham). After blocking with 5% milk (Roth), membranes were incubated with the following antibodies: MIF and CD74 (both Sigma-Aldrich); CDK4 and β-Actin (both Abcam); HSC70 [B-6], total AKT, phospho-AKT [D9E], phospho-p44/42 MAPK (ERK1/2), phospho-p38 MAPK [3D7], p38 MAPK, cleaved caspase-3, and PARP (all Cell Signaling); VEGF, STAT3, and ERK (all Santa Cruz). All primary antibodies were detected with HRP-conjugated secondary antibodies. Development of the signal was performed using Immobilion western chemiluminescent HRP substrate (Millipore/Merck) or Clarity Max™ Western ECL Substrate (BioRad). Detailed antibody information in Supplemental Table 2.

### Quantitative real-time PCR (qRT-PCR)

RNA from human cell lines and colonic tissues and tumors was isolated using Trizol reagent (Invitrogen) according to manufacturer guidelines. Tissues and tumor pieces were shredded using a homogenizer (T10 basic ULTRA-TURRAX). After reverse transcription (M-MuLV Reverse Transcriptase from NEB) of equal amounts of mRNA, quantitative real-time PCR analysis was performed using a qPCR MasterMix (72 mM Tris-HCl pH 8.8 (Roth), 19 mM (NH4)2SO4 (Roth), 0.01% Tween-20 (AppliChem), 3 mM MgCl_2_, (Sigma-Aldrich), 1:80,000 SYBR Green (Invitrogen), 0.24 mM dNTPs, (dATP, dCTP, dGTP, dTTP, all dNTPs from Primetech), 19 U/ml Taq-polymerase (Primetech), 0.24% Triton X-100 (AppliChem), 300 mM Trehalose (Roth). Used primers are listed in Supplemental Table 1. For gene analysis, at least two different cDNAs (technical replicates) were used for qRT-PCR runs from one biological replicate. Biological replicates are independent experiments.

### Quantification and statistical analysis

Statistics of each experiment such as number of animals, number of tumors, biological replicates, technical replicates, precision measures (mean and ±SD), and the statistical tests used for significance are provided in the figures and figure legends.

Densitometric measurements for quantification of immunoblot bands were done with the gel analysis software Image Lab™ (BioRad) and normalized to loading controls.

Pearson correlation factor R was used for analysis of immunohistochemical correlation studies on CRC tissue. GraphPad Prism was used for analysis of Kaplan–Meier survival curves with the Log-rank (Mantel-Cox) test.

The following designations for levels of significance were used within this manuscript: *p ≤ 0.05; **p ≤ 0.01; ***p ≤ 0.001; ns, not significant.

## Supplementary information

Supplemental figure legends

Supp Figure 1

Supp Figure 2

Supp Figure 2

Supp Figure 3

Supp Figure 4

Supp Figure 5

Supplemental Tables 1 and 2
