# Supplementary material for: Hsp90-stabilized MIF supports tumor progression via macrophage recruitment and angiogenesis in colorectal cancer
Source: Cell Death Dis. 2021 Feb 4;12(2):155. doi: 10.1038/s41419-021-03426-z (PMC7862487; doi:10.1038/s41419-021-03426-z)
Supplement: Supplementary file 1 — Supplemental figure legends [file 41419_2021_3426_MOESM1_ESM.docx]

**Figure S1:** Related to Figure 2. **MIF levels are elevated in colorectal cancer cells.**

(A) Pooled cases of single patients (P1-P3), including tumor samples (’T’) and their adjacent normal epithelium (‘N’) (n=3 each). qRT-PCRs normalized to *RPLP0* mRNA. *MIF* expression in tumor samples was calculated relative to respective adjacent epithelium. Mean ± SD of 3 technical replicates in duplicates.

(B) Mif protein level in pooled samples of tumors (‘T’) and normal epithelium (‘N’) of *Mif^+/+^* mice. *Mif^-/-^* tumors serve as negative staining control. Mif ratios (Mif/Actin) were calculated by densitometry, normalized to loading control, relative to *Mif^+/+^* epithelium (‘N’).

(C) Relative *Mif* mRNA levels of pooled normal (‘N’, n≥2) and tumor (‘T’, n=6) samples from *Mif^+/+^* and *Mif^-/-^* mice. Mean ± SD of 5 technical replicates in duplicates.

A, C Student’s t test used for comparison of indicated groups: ***p≤0.001.

**Figure S2:** Related to Figure 3. **A MIF deletion protects mice from inflammation-associated cancer initiation.**

(A) Histological staining of colonic tissues 8 days after DSS (recovery period) of indicated genotypes. Inflammatory marker CD68 (Cluster of Differentiation 68) for monocytes/macrophages, CD3 (Cluster of Differentiation 3) for T-lymphocytes, FoxP3 (Foxhead-box-protein P3) for regulatory T-cells and MPO (Myeloperoxidase) for neutrophils/granulocytes. Scale bars, 100 µm.

(B) Correlation between the inflammatory score of *Mif^+/+^* and *Mif^-/-^* mice (Figure 3B) and the respective quantification of infiltrating immune cells (CD3, MPO and FoxP3 staining) in the recovery group from (Figure 3C). CD3, MPO and FoxP3 with *Mif^+/+^* n=6 and *Mif^-/-^* n=7. R, Pearson correlation factor.

(C) mRNA expression of representative cytokines of individual mice from recovery group (8 days post-DSS) from single samples (4 mice per group). Via qRT-PCR, expression levels were normalized to those of *Rplp0*. Means ± SD of ≥2 technical replicates in duplicates.

(D) Treatment scheme of the AOM/DSS colorectal cancer mouse model. Mice of the ‘short’ group were dissected at day 3 after starting DSS administration.

(E) Representative H&E staining of colonic tissues of indicated genotype at day 3 after DSS start (short, ‘S’) or in untreated control tissue (‘N’). Scale bar, 100 µm.

(F) The inflammatory score of the ‘short’ DSS and control groups was assessed based on H&E stained tissue morphology in (E). Non-treated control n=3 mice per group, short n=6 mice per group. Black line, mean. p value with Student’s t test.

(G) Representative histology of inflammatory cells (CD68, CD3, FoxP3, MPO staining) in *Mif^+/+^* and *Mif^-/-^* colonic tissues at day 3 after DSS start. Scale bars, 100 µm. For quantifications of histological staining, 4-5 images (area=40x magnification) per mouse were counted for positive stained stromal cells. n, number of mice. Black line, mean. p values were calculated via Student’s t test.

(H) A MIF loss is dispensable for AOM-induced DNA damage response. Representative immunohistological phospho-Histone H2A.X staining after the initial AOM injection at indicated time points. Scale bars, 100 µm.

(I) A MIF loss does not impair the AOM-induced p53 response. mRNA level of *Mif* and WTp53 target genes of indicated groups at different time points after a single AOM injection. Single colonic tissues of indicated time points and genotypes were pooled (n≥3 mice per group). qRT-PCRs normalized to *Rplp0* mRNA. Mean ± SD of 2 technical replicates, pipetted in duplicates.

**Figure S3:** Related to Figure 4. **MIF supports CRC tumor growth and macrophage infiltration without affecting overall inflammation.**

(A) Representative histology of CD3, FoxP3 and MPO staining of *Mif^+/+^* and *Mif^-/-^* tumors. Scale bars, 100 µm.

(B) mRNA expression of inflammatory genes in tumors (‘T’) and nontreated control tissues (‘N’) of indicated genotypes. Single colonic tissues (n≥2) or single tumors (n≥6) of indicated genotypes were pooled. qRT-PCR, expression levels were normalized to those of *Rplp0*. Means ± SD of 3-4 technical replicates in duplicate. Student’s t test.

(C) Relative expression of *Vegfa*, averaged from single tumor samples (7-8 mice) (Figure 4C). Means ± SD of 4 technical replicates.

(D) Phospho (p)-Akt level of single tumors (T1-T3) of *Mif^+/+^* and *Mif^-/-^* mice. Hsc70, loading control.

(E) Apoptotic gene expression of indicated tumors. Pooled single tumors (n≥6). qRT-PCR normalized to *Rplp0* mRNA. Mean ± SD of ≥5 technical replicates in duplicates. p value with Student’s t test. ***p≤0.001; ns, not significant.

(F) TUNEL staining in *Mif^+/+^* and *Mif^-/-^* tumors at 12 weeks post-AOM. Scale bars, 100 µm.

(G, H) Representative immunofluorescence of *Mif^+/+^* and *Mif^-/-^* colonic tissue at 8 days post-DSS (recovery group), for CD31 (red) and DAPI (blue). Scale bars, 100 µm. Quantification of tumor vessel fragment density from (G). At least 6 images (area=40x magnification) per colonic tissue were counted and calculated. Black lines, mean. n, number of mice. p value with Student’s t test.

(I) Relative expression of *Vegfa* in recovering *Mif^+/+^* and *Mif^-/-^* colonic tissue (pool of 4-5 mice per group). Via qRT-PCR expression was calculated relative to those of *Rplp0*. Mean ± SD of 3 technical replicates in duplicates. p value with Student’s t test. ns, not significant.

(J, K) Quantification of epithelial Ki67 staining (J) and representative histology of recovering *Mif^+/+^* and *Mif^-/-^* colonic tissue at 8 days post-DSS (K). Ki67 positivity was performed using 6 images per mouse. n, number of mice. Normalization of positive epithelial cells to total number of epithelial cells. p value with Student’s t test. Scale bars, 100 µm.

**Figure S4:** Related to Figure 5. **Knockdown efficiency of MIF and CD74 by small interfering RNA (siRNA) in CRC epithelial cells.**

(A, B) Expression of *MIF* and *CD74* in HCT116 (A) and DLD-1 (B) cells normalized to *RPLP0* or *HPRT1* respectively for evaluation of knockdown efficiency after 72 hrs siRNA transfection against *MIF*, *CD74* or respective scrambled control (‘con’). Mean ± SD of 5-6 technical replicates in duplicates from 2 biological replicates.

Student’s t test: ns=not significant, *p≤0.05; **p≤0.01; ***p≤0.001.

**Figure S5:** Related to Figure 7. **MIF is an actionable and selective therapeutic target by Hsp90 inhibition in colorectal cancer-derived organoids.**

(A) Representative images to evaluate growth and development of normal colonic organoids from two different mouse strains (129S1/SvImJ and C57BL/6). Images were taken at day of organoid preparation (d 0) as well as two (d 2) and six (d 6) days after preparation (p0=passage 0). Additional image taken three (d 3) days after splitting (p1=passage 1). Scale bars, 200 µm.

(B) Representative images of murine normal small intestinal organoids after treatment with DMSO control (‘con’) and indicated concentrations of 17AAG, Ganetespib (‘Ganet’) or Onalespib for 21 hrs. Scale bars, 200 µm. Quantification reveal percentage of dead organoids relative to the total amount of organoids (≥9 images per condition from ≥5 gel domes). Mean ± SD from different images. p values in relation to control (‘con’). *p≤0.05; ***p≤0.001.

(C) Hsp90 inhibitor treatment as in (B) of matched pairs. Immunoblot analysis to evaluate Mif degradation. Well known Hsp90-stabilized protein Stat3 used as positive control for the treatment. Actin, loading control. Mif expression ratios (Mif/Actin) were calculated by densitometry, normalized to the loading control, vehicle control.
