# Supplementary material for: Hsp90-stabilized MIF supports tumor progression via macrophage recruitment and angiogenesis in colorectal cancer
Source: Cell Death Dis. 2021 Feb 4;12(2):155. doi: 10.1038/s41419-021-03426-z (PMC7862487; doi:10.1038/s41419-021-03426-z)
Supplement: Supplementary file 2 — Supp Figure 1 [file 41419_2021_3426_MOESM2_ESM.pptx]

## Slide 1
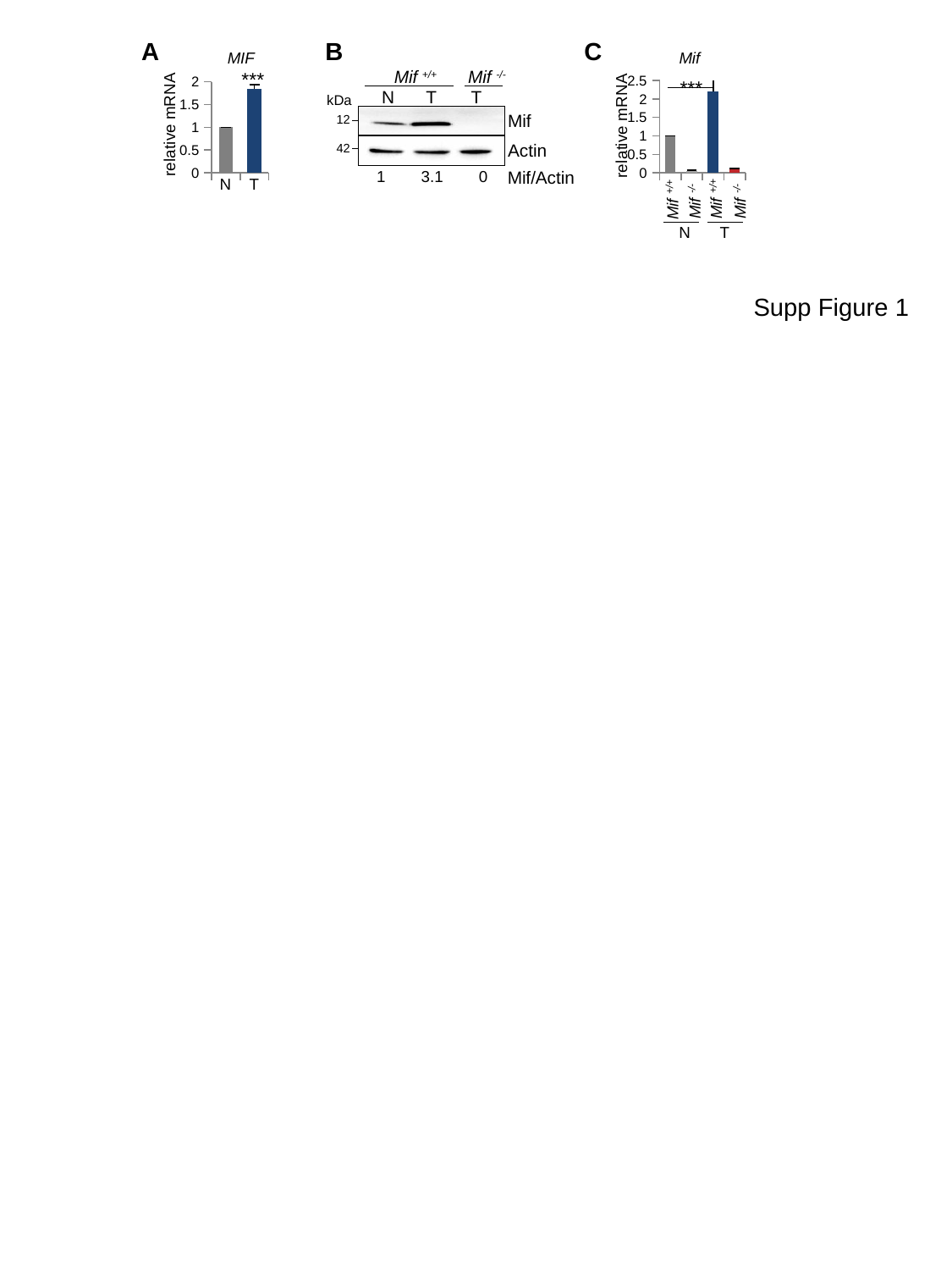

A
B
C
MIF
Mif
Mif +/+
Mif -/-
N
T
T
Mif
12
42
Actin
 1 3.1 0
Mif/Actin
kDa
***
### Chart
| Category | |
|---|---|
| Normal | 1.0 |
| Tumor | 1.8485528194787149 |N
T
### Chart
| Category | MIF |
|---|---|
| +/+ | 1.0 |
| -/- | 0.0585861428995441 |
| +/+ | 2.1929480194706397 |
| -/- | 0.09451205426634884 |***
Mif -/-
Mif +/+
Mif +/+
Mif -/-
N
T
Supp Figure 1
