# Supplementary material for: Hsp90-stabilized MIF supports tumor progression via macrophage recruitment and angiogenesis in colorectal cancer
Source: Cell Death Dis. 2021 Feb 4;12(2):155. doi: 10.1038/s41419-021-03426-z (PMC7862487; doi:10.1038/s41419-021-03426-z)
Supplement: Supplementary file 3 — Supp Figure 2 [file 41419_2021_3426_MOESM3_ESM.pptx]

## Slide 1
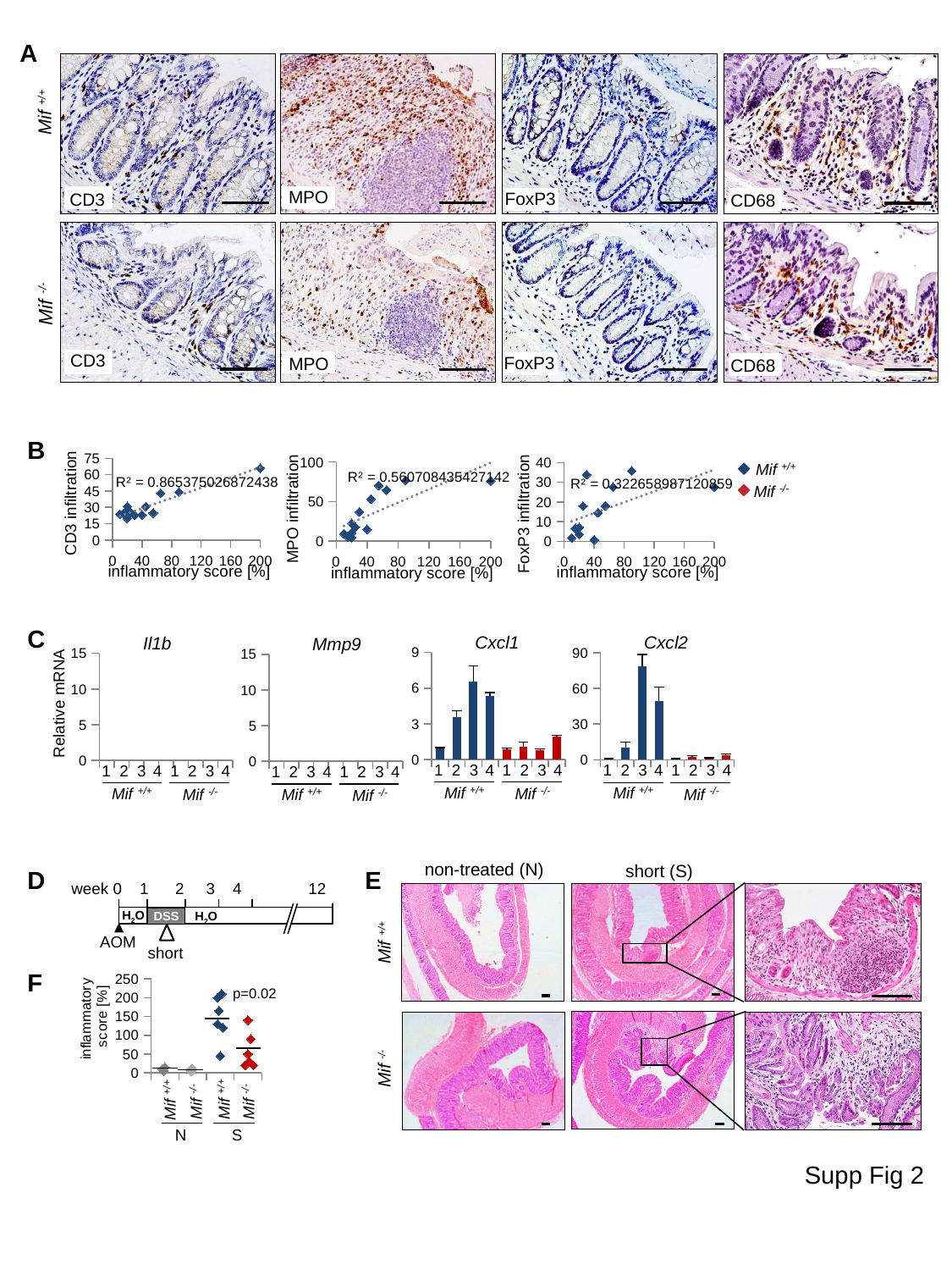

A
Mif +/+
MPO
FoxP3
CD3
CD68
Mif -/-
CD3
FoxP3
MPO
CD68
CD3 infiltration
inflammatory score [%]
### Chart
| Category | |
|---|---|MPO infiltration
inflammatory score [%]
### Chart
| Category | |
|---|---|B
### Chart
| Category | |
|---|---|FoxP3 infiltration
inflammatory score [%]
Mif +/+
Mif -/-
### Chart
| Category | KC |
|---|---|
| 477 | 1.0 |
| 493 | 3.5648048346482586 |
| 683 | 6.573067808574844 |
| 687 | 5.3029373846957855 |
| 480 | 0.8766847207927198 |
| 496 | 1.0945404822886795 |
| 498 | 0.7627905209159879 |
| 483 | 1.9006929844082479 |Cxcl1
1
2
3
4
1
2
3
4
Mif +/+
Mif -/-
### Chart
| Category | CXCL2 |
|---|---|
| 477 | 1.0 |
| 493 | 10.113777959286852 |
| 683 | 78.20252746174731 |
| 687 | 49.35982041869776 |
| 480 | 0.9139859601531422 |
| 496 | 2.4298302894225503 |
| 498 | 1.2202913166308906 |
| 483 | 3.492917762315385 |Cxcl2
1
2
3
4
1
2
3
4
Mif +/+
Mif -/-
### Chart
| Category | Il1b |
|---|---|
| 477 | 1.0 |
| 493 | 4.30745003362897 |
| 683 | 13.66907876834466 |
| 687 | 12.880916170085062 |
| 480 | 0.5277947145135238 |
| 496 | 1.214971733542325 |
| 498 | 0.7445044839549446 |
| 483 | 1.5241376228532717 |Il1b
Relative mRNA
1
2
3
4
1
2
3
4
Mif +/+
Mif -/-
### Chart
| Category | MMP9 |
|---|---|
| 477 | 1.0 |
| 493 | 4.734666476154783 |
| 683 | 26.70545186157848 |
| 687 | 30.524982794386332 |
| 480 | 0.5723195634425511 |
| 496 | 1.19966190956783 |
| 498 | 1.2674080621908728 |
| 483 | 1.0245146847725133 |Mmp9
1
2
3
4
1
2
3
4
Mif +/+
Mif -/-
C
non-treated (N)
short (S)
Mif +/+
Mif -/-
D
E
### Chart
| Category | | -/- | +/+ | -/- |
|---|---|---|---|---|p=0.02
Mif +/+
Mif -/-
Mif +/+
Mif -/-
N
S
week 0 1 2 3 4 12
H2O
H2O
DSS
AOM
short
F
Supp Fig 2
