# Supplementary material for: Hsp90-stabilized MIF supports tumor progression via macrophage recruitment and angiogenesis in colorectal cancer
Source: Cell Death Dis. 2021 Feb 4;12(2):155. doi: 10.1038/s41419-021-03426-z (PMC7862487; doi:10.1038/s41419-021-03426-z)
Supplement: Supplementary file 4 — Supp Figure 2 [file 41419_2021_3426_MOESM4_ESM.pptx]

## Slide 1
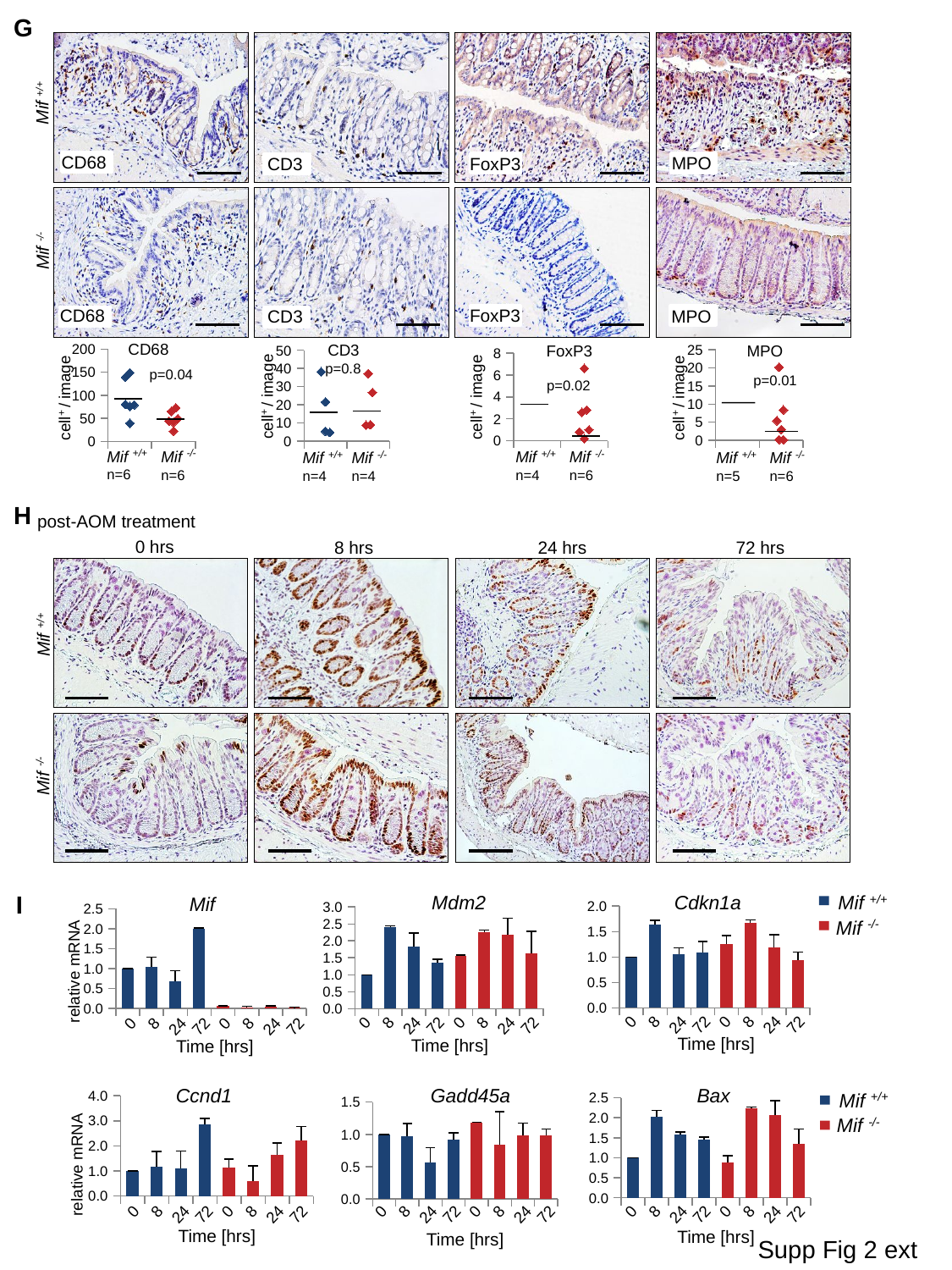

G
Mif +/+
CD68
MPO
CD3
FoxP3
Mif -/-
FoxP3
CD68
MPO
CD3
### Chart
| Category | | |
|---|---|---|CD3
p=0.8
cell+ / image
Mif +/+
n=4
Mif -/-
n=4
### Chart
| Category | | |
|---|---|---|FoxP3
p=0.02
cell+ / image
Mif +/+
n=4
Mif -/-
n=6
### Chart
| Category | | |
|---|---|---|
### Chart
| Category | | |
|---|---|---|MPO
p=0.01
cell+ / image
Mif +/+
n=5
Mif -/-
n=6
CD68
p=0.04
cell+ / image
Mif +/+
n=6
Mif -/-
n=6
H
post-AOM treatment
0 hrs
8 hrs
24 hrs
72 hrs
Mif +/+
Mif -/-
### Chart
| Category | Bax |
|---|---|
| +/+ | 1.0 |
| +/+ | 2.39518754582458 |
| +/+ | 1.823682212896183 |
| +/+ | 1.3451857742861713 |
| -/- | 1.56737320258258 |
| -/- | 2.2531523557413937 |
| -/- | 2.1891389046996745 |
| -/- | 1.6252291100527347 |
### Chart
| Category | Bax |
|---|---|
| +/+ | 1.0 |
| +/+ | 1.0363801604164022 |
| +/+ | 0.6876543850857222 |
| +/+ | 2.0069555500567224 |
| -/- | 0.06180286826790285 |
| -/- | 0.027310075476679498 |
| -/- | 0.05618194843793025 |
| -/- | 0.03193422625062949 |
### Chart
| Category | Bax |
|---|---|
| +/+ | 1.0 |
| +/+ | 1.631329410259279 |
| +/+ | 1.0570069887668754 |
| +/+ | 1.091767201256241 |
| -/- | 1.2498736048929184 |
| -/- | 1.6706673580852276 |
| -/- | 1.195298539893599 |
| -/- | 0.947128725604277 |Mif +/+
I
Cdkn1a
Mdm2
Mif
Mif -/-
relative mRNA
0
8
0
8
24
72
24
72
Time [hrs]
0
8
0
8
0
8
0
8
24
72
24
72
24
72
24
72
### Chart
| Category | Bax |
|---|---|
| +/+ | 1.0 |
| +/+ | 1.1772275279842503 |
| +/+ | 1.0870108209149765 |
| +/+ | 2.8723208939580758 |
| -/- | 1.1329039379538406 |
| -/- | 0.5915585350192853 |
| -/- | 1.629149447053925 |
| -/- | 2.2260474377137958 |
### Chart
| Category | Bax |
|---|---|
| +/+ | 1.0 |
| +/+ | 2.026221981873455 |
| +/+ | 1.5763208938220128 |
| +/+ | 1.444796457492671 |
| -/- | 0.8872885806067365 |
| -/- | 2.242386023034819 |
| -/- | 2.061954376994548 |
| -/- | 1.3428420822645766 |Time [hrs]
Time [hrs]
### Chart
| Category | Bax |
|---|---|
| +/+ | 1.0 |
| +/+ | 0.9752329436607778 |
| +/+ | 0.5679686439695214 |
| +/+ | 0.9140902074327029 |
| -/- | 1.1789638600867274 |
| -/- | 0.8356987120669271 |
| -/- | 0.9805514671011184 |
| -/- | 0.9883716733421143 |Ccnd1
Gadd45a
Bax
Mif +/+
Mif -/-
relative mRNA
0
8
0
8
0
8
0
8
0
8
0
8
24
72
24
72
24
72
24
72
24
72
24
72
Time [hrs]
Time [hrs]
Time [hrs]
Supp Fig 2 ext
