# Supplementary material for: Hsp90-stabilized MIF supports tumor progression via macrophage recruitment and angiogenesis in colorectal cancer
Source: Cell Death Dis. 2021 Feb 4;12(2):155. doi: 10.1038/s41419-021-03426-z (PMC7862487; doi:10.1038/s41419-021-03426-z)
Supplement: Supplementary file 5 — Supp Figure 3 [file 41419_2021_3426_MOESM5_ESM.pptx]

## Slide 1
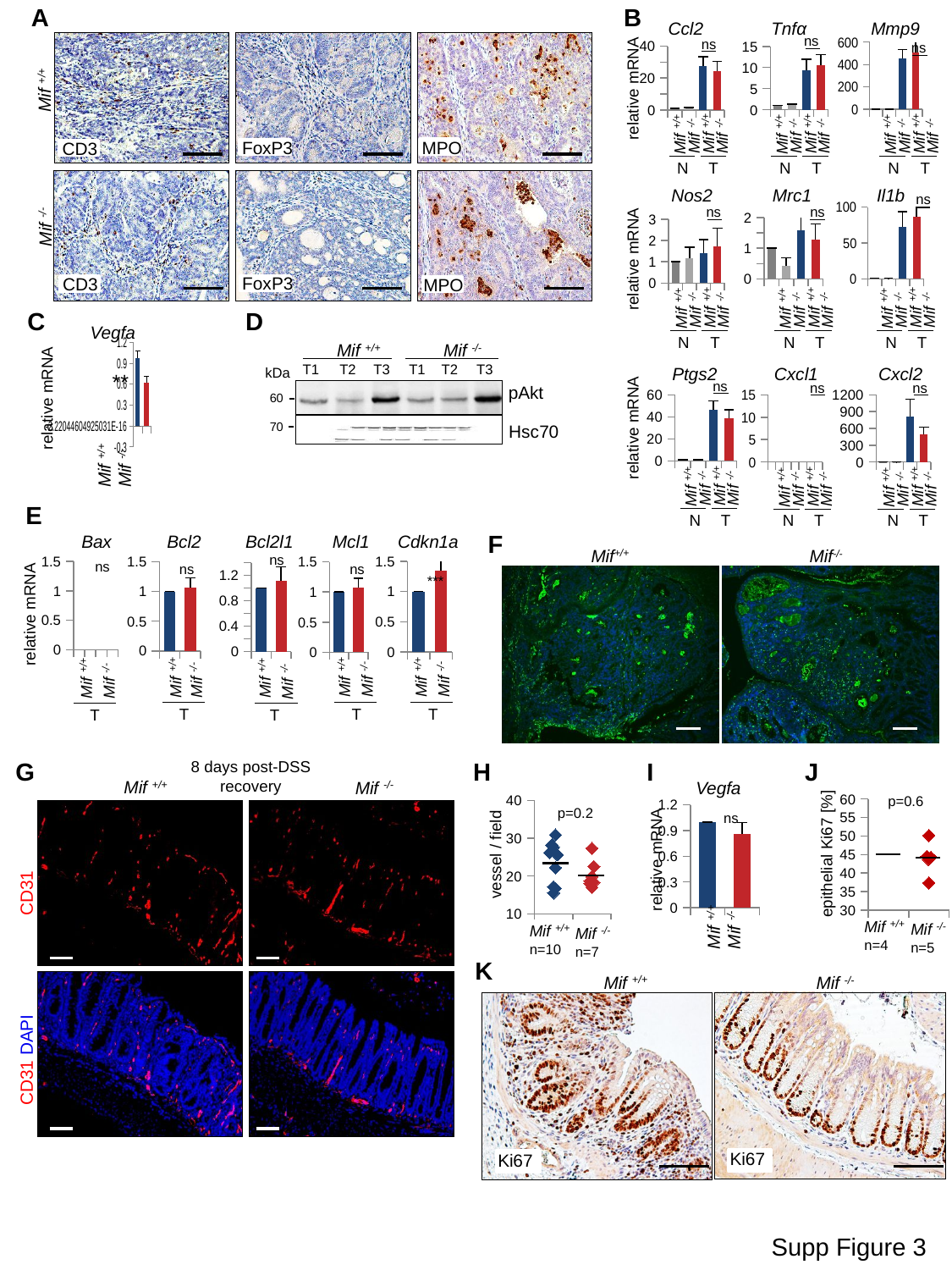

A
B
### Chart
| Category | CCL2 |
|---|---|
| +/+ | 1.0 |
| -/- | 1.084443959360307 |
| +/+ | 27.63087670260099 |
| -/- | 24.441463078346796 |
### Chart
| Category | MMP9 |
|---|---|
| +/+ | 1.0 |
| -/- | 0.8173591960391963 |
| +/+ | 457.06771517155425 |
| -/- | 509.13050969749855 |Ccl2
Tnfα
Mmp9
ns
ns
Mif +/+
FoxP3
MPO
CD3
Mif -/-
FoxP3
CD3
MPO
ns
### Chart
| Category | TNFa |
|---|---|
| +/+ | 1.0 |
| -/- | 1.1835680273098952 |
| +/+ | 9.433011307075375 |
| -/- | 10.523465293032586 |relative mRNA
Mif +/+
Mif -/-
Mif +/+
Mif -/-
T
N
Mif +/+
Mif -/-
Mif +/+
Mif -/-
T
N
Mif +/+
Mif -/-
Mif +/+
Mif -/-
N
T
### Chart
| Category | MRC1 |
|---|---|
| +/+ | 1.0 |
| -/- | 0.4115537825300104 |
| +/+ | 1.5764977238526292 |
| -/- | 1.2895582660577007 |Nos2
Mrc1
Il1b
ns
### Chart
| Category | iNOS |
|---|---|
| +/+ | 1.0 |
| -/- | 1.1704031337865686 |
| +/+ | 1.408999875522559 |
| -/- | 1.7358473520619133 |
### Chart
| Category | IL-1b |
|---|---|
| +/+ | 1.0 |
| -/- | 0.6507852538563735 |
| +/+ | 72.35281175996646 |
| -/- | 86.49715157092244 |ns
ns
relative mRNA
Mif +/+
Mif -/-
Mif +/+
Mif -/-
T
N
Mif +/+
Mif -/-
Mif +/+
Mif -/-
N
T
Mif +/+
Mif -/-
Mif +/+
Mif -/-
N
T
C
D
Vegfa
**
Mif +/+
Mif -/-
relative mRNA
[unsupported chart]
Mif +/+
Mif -/-
T1
T2
T3
T1
T2
T3
pAkt
60
70
Hsc70
kDa
Ptgs2
Cxcl1
Cxcl2
### Chart
| Category | CXCL-1 |
|---|---|
| +/+ | 1.0 |
| -/- | 0.7614540826694044 |
| +/+ | 31.077475310523496 |
| -/- | 29.13836326833955 |
### Chart
| Category | CXCL-2 |
|---|---|
| +/+ | 1.0 |
| -/- | 1.3552453273638345 |
| +/+ | 817.2933231325804 |
| -/- | 486.69605835105517 |ns
ns
ns
### Chart
| Category | COX2 |
|---|---|
| +/+ | 1.0 |
| -/- | 0.9379852731711936 |
| +/+ | 46.37442491727944 |
| -/- | 38.92194785562504 |relative mRNA
Mif +/+
Mif -/-
Mif +/+
Mif -/-
T
N
Mif +/+
Mif -/-
Mif +/+
Mif -/-
T
N
Mif +/+
Mif -/-
Mif +/+
Mif -/-
T
N
### Chart
| Category | Bax |
|---|---|
| CRC +/+ | 1.0 |
| CRC -/- | 1.0476893797757763 |E
Bax
Bcl2
Bcl2l1
Mcl1
Cdkn1a
ns
ns
ns
ns
***
relative mRNA
Mif +/+
Mif -/-
T
Mif +/+
Mif -/-
T
Mif +/+
Mif -/-
T
Mif +/+
Mif -/-
T
Mif +/+
Mif -/-
T
### Chart
| Category | Mcl1 |
|---|---|
| CRC +/+ | 1.0 |
| CRC -/- | 1.0669803668942435 |
### Chart
| Category | BclXL |
|---|---|
| CRC +/+ | 1.0 |
| CRC -/- | 1.1186578767119502 |
### Chart
| Category | Bcl2 |
|---|---|
| CRC +/+ | 1.0 |
| CRC -/- | 1.0713581986086176 |
### Chart
| Category | p21 |
|---|---|
| CRC +/+ | 1.0 |
| CRC -/- | 1.3438695812152102 |F
Mif+/+
Mif-/-
G
H
I
J
8 days post-DSS recovery
Mif +/+
Mif -/-
CD31
CD31 DAPI
Vegfa
ns
Mif +/+
Mif -/-
relative mRNA
### Chart
| Category | VEGFA |
|---|---|
| +/+ | 1.0 |
| -/- | 0.856008621109873 |
### Chart
| Category | | |
|---|---|---|p=0.2
Mif +/+
n=10
Mif -/-
n=7
vessel / field
epithelial Ki67 [%]
Mif +/+
n=4
Mif -/-
n=5
p=0.6
### Chart
| Category | | |
|---|---|---|K
Mif +/+
Mif -/-
Ki67
Ki67
Supp Figure 3
