# Supplementary material for: Hsp90-stabilized MIF supports tumor progression via macrophage recruitment and angiogenesis in colorectal cancer
Source: Cell Death Dis. 2021 Feb 4;12(2):155. doi: 10.1038/s41419-021-03426-z (PMC7862487; doi:10.1038/s41419-021-03426-z)
Supplement: Supplementary file 6 — Supp Figure 4 [file 41419_2021_3426_MOESM6_ESM.pptx]

## Slide 1
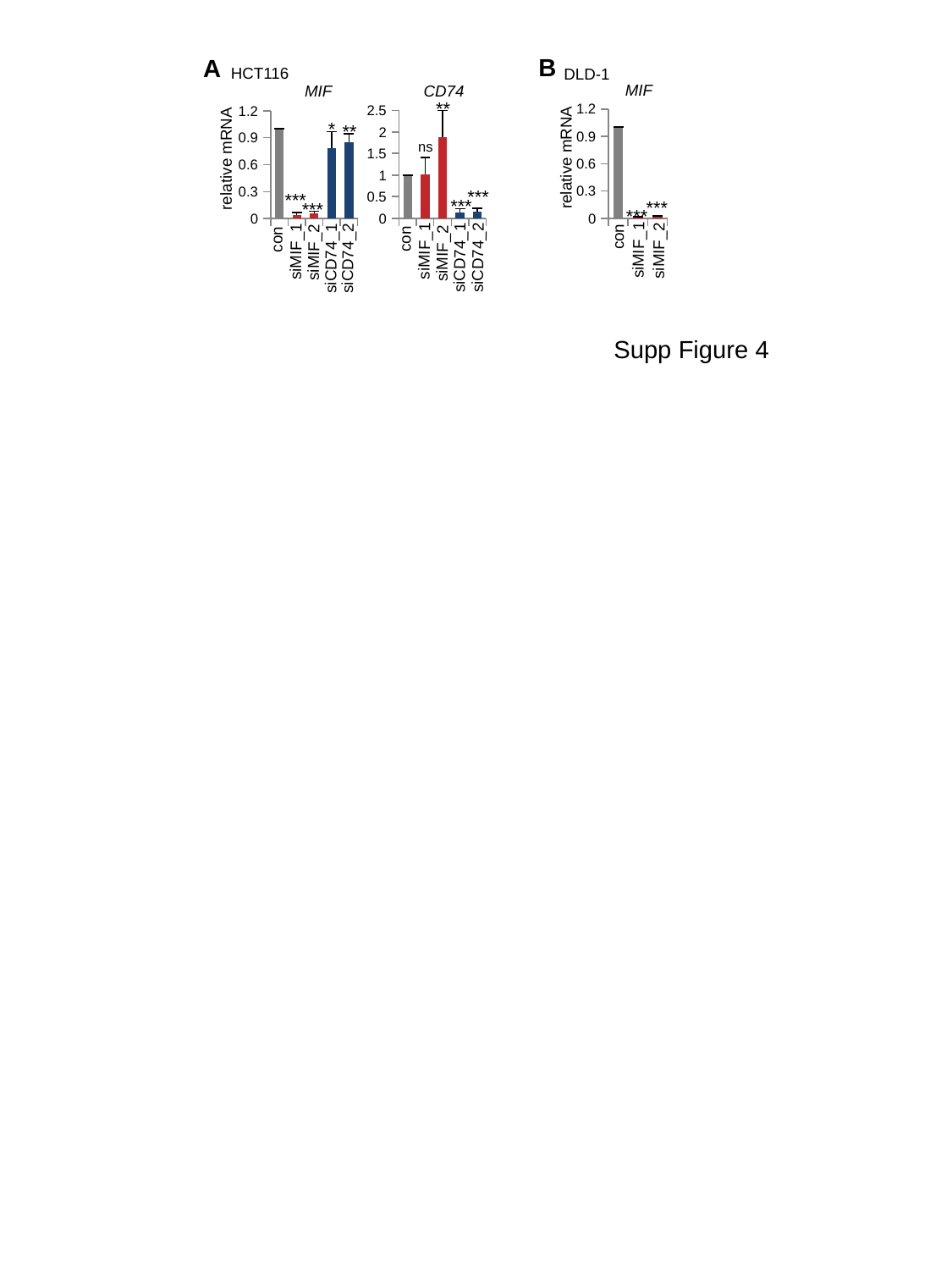

### Chart
| Category | CD74 |
|---|---|
| | 1.0 |
| 1 | 1.0115416362018996 |
| 3 | 1.8703019927568298 |
| 5 | 0.12491077950534521 |
| 9 | 0.15255695262772523 |
### Chart
| Category | MIF |
|---|---|
| | 1.0 |
| 1 | 0.04425815386465655 |
| 3 | 0.05588201256411722 |
| 5 | 0.787423496365584 |
| 9 | 0.8491783255419325 |
### Chart
| Category | MIF |
|---|---|
| ssc2 | 1.0 |
| siMIF#1 | 0.014261865779767758 |
| siMIF#3 | 0.021659745156153012 |MIF
relative mRNA
con
siMIF_1
siMIF_2
B
A
HCT116
MIF
CD74
**
*
ns
relative mRNA
***
***
***
***
con
con
siMIF_1
siMIF_1
siMIF_2
siMIF_2
siCD74_1
siCD74_2
siCD74_1
siCD74_2
DLD-1
**
***
***
Supp Figure 4
