# Supplementary material for: Hsp90-stabilized MIF supports tumor progression via macrophage recruitment and angiogenesis in colorectal cancer
Source: Cell Death Dis. 2021 Feb 4;12(2):155. doi: 10.1038/s41419-021-03426-z (PMC7862487; doi:10.1038/s41419-021-03426-z)
Supplement: Supplementary file 7 — Supp Figure 5 [file 41419_2021_3426_MOESM7_ESM.pptx]

## Slide 1
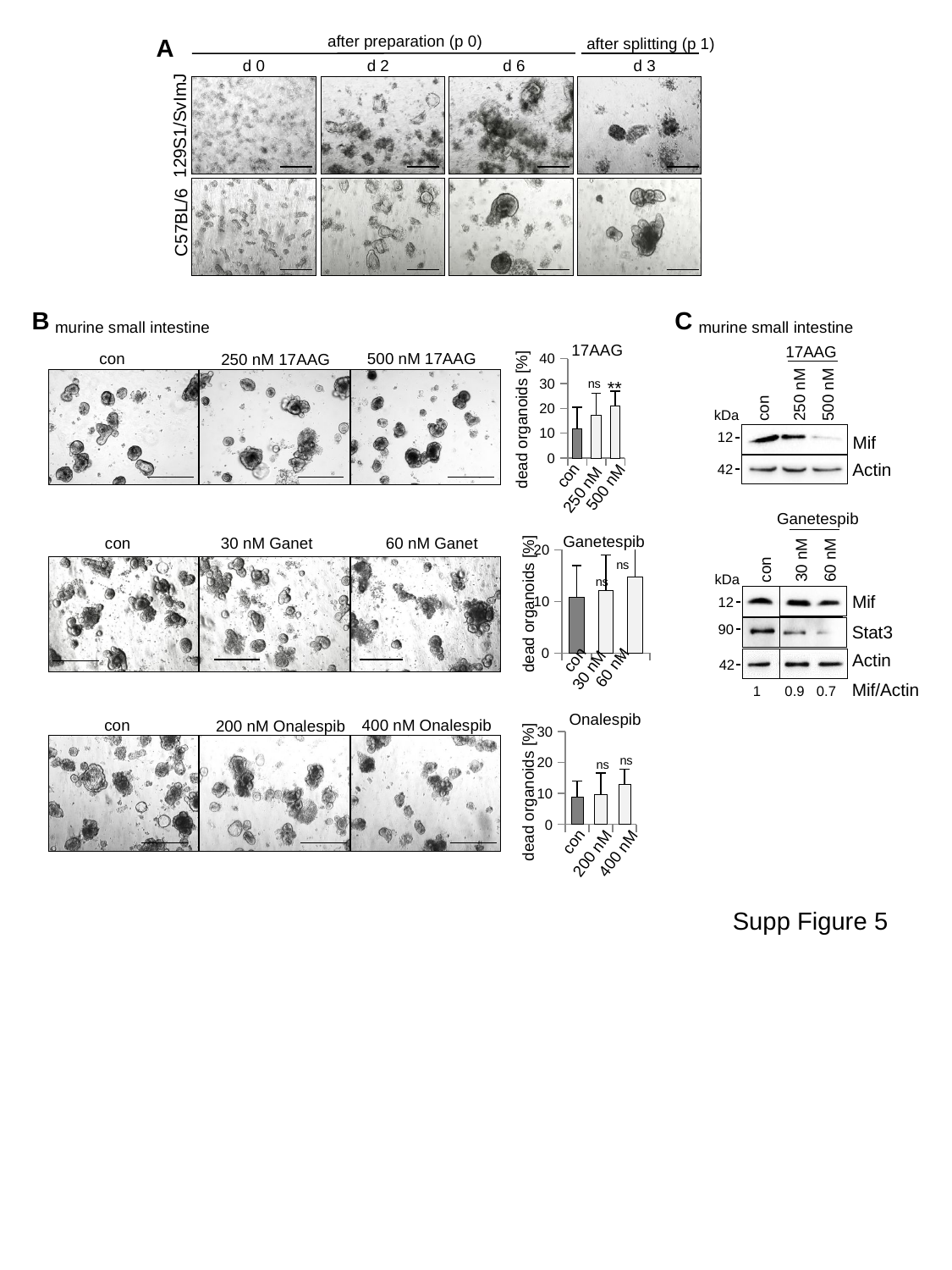

after preparation (p 0)
A
after splitting (p 1)
d 0
d 2
d 6
d 3
129S1/SvImJ
C57BL/6
B
C
murine small intestine
murine small intestine
### Chart
| Category | SI |
|---|---|
| DMSO | 11.857537361923326 |
| 0.25µM 17-AAG | 17.10613393584408 |
| 0.5µM 17-AAG | 20.9848783827112 |17AAG
ns
**
dead organoids [%]
con
500 nM
250 nM
17AAG
250 nM
500 nM
con
kDa
12
Mif
Actin
42
500 nM 17AAG
con
250 nM 17AAG
Ganetespib
30 nM
60 nM
con
kDa
Mif
12
90
Stat3
Actin
42
Mif/Actin
1 0.9 0.7
dead organoids [%]
con
60 nM
30 nM
Ganetespib
con
30 nM Ganet
60 nM Ganet
### Chart
| Category | NORMAL |
|---|---|
| DMSO | 10.880740454076365 |
| 30 ganet | 12.1896074198706 |
| 60 ganet | 14.692307692307693 |ns
ns
### Chart
| Category | |
|---|---|
| DMSO | 8.827561327561327 |
| 200nM Onalespib | 9.482790107313372 |
| 400nM Onalespib | 13.016475383348446 |Onalespib
ns
ns
dead organoids [%]
con
200 nM
400 nM
400 nM Onalespib
con
200 nM Onalespib
Supp Figure 5
