# Supplementary material for: Hsp90-stabilized MIF supports tumor progression via macrophage recruitment and angiogenesis in colorectal cancer
Source: Cell Death Dis. 2021 Feb 4;12(2):155. doi: 10.1038/s41419-021-03426-z (PMC7862487; doi:10.1038/s41419-021-03426-z)
Supplement: Supplementary file 8 — Supplemental Tables 1 and 2 [file 41419_2021_3426_MOESM8_ESM.docx]

Supplemental Table 1: Primers

| **Gene** | **Origin** | **Forward** | **Reverse** |
| --- | --- | --- | --- |
| **Quantitative RT-PCR** | | | |
| *RPLP0 (36B4)* | Human | 5’-GATTGGCTACCCAACTGTTG | 5’-CAGGGGCAGCAGCCACAAA |
| *HPRT1* | Human | 5’-CCTGGCGTCGTGATTAGTGAT | 5’-GGGCTACAATGTGATGGCCT |
| *MIF_1* | Human | 5’-AGCAGCTGGCGCAGGCCAC | 5’-CTCGCTGGAGCCGCCGAAGG |
| *MIF_2* | Human | 5’-GCAGCTGGCGCAGGCCAC | 5’-GGAGCCGCCGAAGGCCATGA |
| *CD74* | Human | 5’-GGCAACATGACAGAGGACCA | 5’-AGTGACTCTTTCGGTGGAGC |
| *CXCL8 (IL8)_1* | Human | 5’-GCTCTGTGTGAAGGTGCAGTT | 5’-AATTTCTGTGTTGGCGCAGT |
| *CXCL8 (IL8)_2* | Human | 5’-ACACTGCGCCAACACAGAAA | 5’-TTGCTTGAAGTTTCACTGGCAT |
| *VEGFA* | Human | 5’-CTCCACCATGCCAAGTGGT | 5’-GTCCACCAGGGTCTCGATTG |
| *Rplp0 (36B4)* | Mouse | 5’-GCAGATCGGGTACCCAACTGTT | 5’-CAGCAGCCGCAAATGCAGATG |
| *Mif_1* | Mouse | 5’-TCCGTGCCAGAGGGGTTTCTGT | 5’-ACGTTGGCAGCGTTCATGTCG |
| *Mif_2* | Mouse | 5’-CTCCGTGCCAGAGGGGTTTCT | 5’-GCACCACCGATCTTGCCGATG |
| *Arg1* | Mouse | 5’-GAGCATGAGCTCCAAGCCAA | 5’-TCTCTCACGTCATACTCTGTTTCT |
| *Bax* | Mouse | 5’-GCTGATGGCAACTTCAACTGG | 5’-TGATCAGCTCGGGCACTTTAG |
| *Bcl2* | Mouse | 5’-GACTGAGTACCTGAACCGGC | 5’-AGTTCCACAAAGGCATCCCAG |
| *Bcl2l1* | Mouse | 5’-TCGCCGGAGATAGATTTGAATAACC | 5’-TGGGCTCAACCAGTCCATTG |
| *Ccl2* | Mouse | 5’-GTCCCTGTCATGCTTCTGGG | 5’-GAGTAGCAGCAGGTGAGTGG |
| *Ccl5* | Mouse | 5’-TCACCATATGGCTCGGACA | 5’-TTCTCTGGGTTGGCACACAC |
| *Ccnd1* | Mouse | 5’-GGAGCTGCTGCAAATGGAAC | 5’-CAGTCCGGGTCACACTTGA |
| *Cdkn1a (p21)* | Mouse | 5’-GTGGCCTTGTCGCTGTCTT | 5’-GCGCTTGGAGTGATAGAAATCTG |
| *Clec7a_1* | Mouse | 5’-AGGAAGCCGGGCTCCAT | 5’-TACCACAAAGCACAGGATTCCTAAA |
| *Clec7a_2* | Mouse | 5’-TGGGTGCCCTAGGAGGTTT | 5’-AACCATGGCCCTTCACTCTG |
| *Cxcl1 (Kc)* | Mouse | 5’-GCTGGGATTCACCTCAAGAA | 5’-CTTGGGGACACCTTTTAGCA |
| *Cxcl2* | Mouse | 5’-CTCTCAAGGGCGGTCAAAAAG | 5’-TTGGTTCTTCCGTTGAGGGAC |
| *Gadd45a* | Mouse | 5’-AAGCTGCTCAACGTAGACCCC | 5’-ATCCATGTAGCGACTTTCCCG |
| *Il1b* | Mouse | 5’-AGCTTCCTTGTGCAAGTGTCT | 5’-GACAGCCCAGGTCAAAGGTT |
| *Mcl1* | Mouse | 5’-TAAGGACGAAACGGGACTGG | 5’-AGTTTGGTGGCTGGAGCTTTA |
| *Mdm2* | Mouse | 5’-GGTCCCTGTCCTTTGATCCG | 5’-GCTCACTTACGCCATCGTCA |
| *Mmp9* | Mouse | 5’-TCTGTCCAGACCAAGGGTACA | 5’-GCCTTGGGTCAGGCTTAGAG |
| *Mrc1* | Mouse | 5’-TTGGTGGCAATTCACGAGAG | 5’-GGGAAGGGTCAGTCTGTGTTTG |
| *Nos2* | Mouse | 5’-CCCTCCTGATCTTGTGTTGGA | 5’-CAACCCGAGCTCCTGGAAC |
| *Ptgs2* | Mouse | 5’-TCCTGACCCACTTCAAGGGA | 5’-CTCCTTATTTCCCTTCACACCCA |
| *Tnfa* | Mouse | 5’-AGGGATGAGAAGTTCCCAAATG | 5’-TGTGAGGGTCTGGGCCATA |
| *Vegfa_1* | Mouse | 5‘-ACTGGACCCTGGCTTTACTG | 5‘-GATCCGCATGATCTGCATGG |
| *Vegfa_2* | Mouse | 5‘-CTGGACCCTGGCTTTACTGC | 5‘-TGAACTTGATCACTTCATGGGACT |
| **Genotyping** | | | |
| *Mif A1* | Mouse | 5’-AGGTTAGTCACTCTACTGGCC |  |
| *Mif B1* | Mouse | 5’-TCTCACTGTTCTGGTGTGAGG |  |
| *Mif C1* | Mouse | 5’-GGCTCCTGGTCTCAGTCAGG |  |
| *Vil Cre* | Mouse | 5’-CGCGAACATCTTCAGGTTCT |  |
| *Vil Cre* | Mouse | 5’-CAAGCCTGGCTCGACGGCC |  |

Supplemental Table 2: Reagents and Resources

| **REAGENT or RESOURCE** | **SOURCE** | **IDENTIFIER (Cat#)** |
| --- | --- | --- |
| **Antibodies** | | |
| Rabbit polyclonal anti-Akt | Cell Signaling | 9272, RRID:AB_329827 |
| Rabbit monoclonal anti-phospho-Akt (Ser473) [D9E] | Cell Signaling | 4060, RRID:AB_2315049 |
| Mouse polyclonal anti-beta-Actin | Abcam | ab6276, RRID:AB_2223210 |
| Rabbit polyclonal anti-beta-Actin | Abcam | ab8227, RRID:AB_2305186 |
| Rabbit monoclonal anti-CD3 [SP7] | Abcam | ab16669, RRID:AB_443425 |
| Rat monoclonal anti-CD31 [SZ31] | Dianova | DIA-310, RRID:AB_2631039 |
| Rat monoclonal anti-CD68 [FA-11] | Abcam | ab53444, RRID:AB_869007 |
| Rat monoclonal anti-CD68 [FA-11] | eBioscience™ | 14-0681-82, RRID:AB_2572857 |
| Rabbit polyclonal anti-CD74 | Sigma-Aldrich | HPA010592, RRID:AB_1078482 |
| Rabbit monoclonal anti-Cleaved Caspase-3 [5A1E] | Cell Signaling | 9664, RRID:AB_2070042 |
| Rabbit polyclonal anti-FoxP3 | Abcam | ab54501, RRID:AB_880110 |
| Rabbit monoclonal anti-Histone H2A.X, phospho (Ser139) [20E3] | Cell signaling | 9718, RRID:AB_2118009 |
| Mouse monoclonal HSC70 [B-6] | Santa Cruz | sc-7298, RRID:AB_627761 |
| Rabbit polyclonal anti-Ki67 | Abcam | ab15580, RRID:AB_443209 |
| Rabbit polyclonal anti-p38 MAPK | Cell Signaling | 9212, RRID:AB_330713 |
| Rabbit monoclonal anti-phospho-p38 MAPK (Thr180/Tyr182) [3D7] | Cell Signaling | 9215, RRID:AB_331762 |
| Rabbit polyclonal anti-ERK | Santa Cruz | sc-94, RRID:AB_2140110 |
| Rabbit monoclonal anti-phospho-p44/42 MAPK (Erk1/2) (Thr202/Tyr204) [D13.14.4E] | Cell Signaling | 4370, RRID:AB_2315112 |
| Rabbit polyclonal anti-MIF | Sigma-Aldrich | HPA003868, RRID:AB_1079290 |
| Goat polyclonal anti-MPO | R&D system | AF3667, RRID:AB_2250866 |
| Rabbit polyclonal anti-PARP | Cell Signaling | 9542, RRID:AB_2160739 |
| Rabbit polyclonal anti-STAT3 | Santa Cruz | sc-482, RRID:AB_632440 |
| Mouse monoclonal anti-VEGF [C1] | Santa Cruz | sc-7269, RRID:AB_628430 |
| donkey anti-rat IgG (H+L) Alexa Fluor 594 | Invitrogen | A-21209, RRID:AB_2535795 |
| ExtrAvidin®−Peroxidase | Sigma-Aldrich | E2886, RRID:AB_2620165 |
| biotinylated goat/sheep antibody | GE Healthcare | RPN1025-2ML, RRID:AB_1082105 |
| ImmPRESS™ Reagent Anti-Rabbit IgG | Vector Laboratories | MP-7401, RRID:AB_2336529 |
| ImmPRESS™ Reagent Anti-Rat IgG, mouse adsorbed | Vector Laboratories | MP-7444, RRID:AB_2336530 |
| goat anti-rabbit IgG-HRP | Santa Cruz | sc-2004, RRID:AB_631746 |
| goat anti-mouse IgG-HRP | Santa Cruz | sc-2005, RRID:AB_631736 |
| **Bacterial strains** | | |
| Bacteria: ElectroMAX DH10B cells | Invitrogen/Thermo Fisher Scientific | 18290-015 |
| **Chemicals, Peptides and Recombinant Proteins** | | |
| 0.9% sodium chloride | B. Braun | 2001675 |
| 17AAG | Provided by NCI | N/A |
| 3,3'-Diaminobenzidine tetrahydrochloride (DAB) | Roth | CN75.2 |
| Ammonium sulfate (NH_4_)_2_SO_4_ | Roth | 9218.1 |
| AOM (Azoxymethane) | Sigma-Aldrich | A5486 |
| BCA protein assay | Pierce | 23227 |
| Clarity Max™ Western ECL Substrate | BioRad | 1705062 |
| cOmplete^TM^ mini protease inhibitor cocktail | Roche | 11836170001 |
| Cycloheximide | Sigma-Aldrich | C7698 |
| DAPI | Sigma-Aldrich | D9542 |
| dATP | Primetech | 1202.4 |
| dCTP | Primetech | 1203.4 |
| Dextrose | Sigma-Aldrich | D9434 |
| dGTP | Primetech | 1204.4 |
| DirectPCR lysis Reagent | Peqlab | 31-101-T |
| DSS (Dextran sodium sulfate) | MP Biomedicals | 160110 |
| dTTP | Primetech | 1205.4 |
| EDTA | Roth | 8040.1 |
| Eosin G | Roth | 7089.1 |
| Fluorescent Mounting Medium | DakoCytomation | S302380-2 |
| Ganetespib | Provided by Synta Pharmaceuticals | N/A |
| Onalespib (AT13387) | Selleckem | S1163 |
| HRP substrate | Millipore/Merck | WBKLS0500 |
| Imidazole | Roth | 3899.2 |
| Isoflurane CP | CP-Pharma | 1214 |
| Kolliphor^®^ RH 40 | Merck | 07076 |
| Lipofectamine™ 3000 Transfection Reagent | Invitrogen | L3000015 |
| M-MuLV Reverse Transcriptase | New England Biolabs | M0253S |
| Magnesium chloride (MgCl_2_) | Sigma-Aldrich | M1028 |
| Mayer’s Hemalum solution | Merck | 109249 |
| Milk powder | Roth | T145.4 |
| NaCl | Roth | 3957.2 |
| Sodium Fluoride | Applichem | A0401 |
| nitrocellulose membranes | Amersham | GE10600001 |
| OneTaq® Quick-Load® 2X Master Mix | New England Biolabs | M0486L |
| Phusion® High-Fidelity DNA Polymerase | ThermoFisher Scentific | F530 |
| Recombinant human Macrophage migration inhibitory factor | Immunotools | 11344263 |
| Roti ® Histokitt II | Roth | T160.1 |
| SDS | Roth | CN30.3 |
| Sodium deoxycholate | Sigma-Aldrich | 30970 |
| Sodium orthovanadate | Sigma-Aldrich | S6508 |
| SYBR Green | Invitrogen | S7567 |
| Tamoxifen (TAM) | Sigma-Aldrich | T5648 |
| Taq-polymerase | Primetech | 1800.4 |
| Trehalose | Roth | 5151.3 |
| Tris-HCl | Roth | 4855.3 |
| Triton X-100 | AppliChem | A1388 |
| TRIzol^TM^ Reagent | Invitrogen | 15596026 |
| TUNEL enzyme solution | Sigma-Aldrich | 11767305001 |
| TUNEL Label mix | Sigma-Aldrich | 11767291910 |
| Tween-20 | AppliChem | A4974 |
| **Reagents for Cell culture** | | |
| A83-01 | Sigma-Aldrich | SML0788 |
| Advanced DMEM/F12 medium | Gibco | 12634010 |
| B-27 | Gibco | 17504044 |
| Cell Recovery solution | Corning | 11543560 |
| CHIR 99021 | Axon Medchem | 1386 |
| Collagenase type I, powder | Gibco | 17018029 |
| DMEM | Gibco | 61965059 |
| FBS | Merck | S0615 |
| Geneticin (G418) | InvivoGen | ant-gn-1 |
| GlutaMAX™ | Gibco | 35050061 |
| HEPES | Gibco | 15630080 |
| L-Glutamine | Gibco | 25030123 |
| Matrigel | Corning | 354230 |
| McCoy's 5A modified medium | Gibco | 16600082 |
| Mycoplasma Detection Kit | Lonza | LT07-318 |
| N-2 | Gibco | 17502048 |
| N-Acetyl-L-Cysteine | Sigma-Aldrich | A9165 |
| Nicotinamide | Sigma-Aldrich | N3376 |
| Penicillin-Streptomycin | Gibco | 15140122 |
| rmEGF | ImmunoTools | 12343406 |
| ROCK inhibitor (Y-27632) | Sigma-Aldrich | SCM075 |
| RPMI 1640 | Gibco | 42401042 |
| Sodium Pyruvate | Gibco | 11360039 |
| Zeocin | InvivoGen | ant-zn-05 |
| **Experimental models: Cell lines** | | |
| HEK293T Wnt3a | Robyn Laura Kosinsky |  |
| HEK293T Noggin | Farin et. al, 2012 | PMID: 22922422 |
| HEK293T R-spondin I | Farin et. al, 2012 | PMID: 22922422 |
| HCT116 | ATCC | ATCC^®^ CCL-247™ |
| DLD-1 | DSMZ | ACC 278 |
| **Experimental models: Mouse strains** |  |  |
| *Mif* knock-out (129S1/SvImJ) | Fingerle-Rowson et al., 2003 | PMID: 12878730 |
| Mif^fl/fl^ (C57BL/6N) | Fingerle-Rowson et al., 2003, Brocks et al., 2017 | PMID: 12878730, PMID: 27825106 |
| Mouse: villin:CreERT2 | N/A | RRID:IMSR_JAX:020282 |
| C57BL/6NCrl | Charles River | RRID:IMSR_CRL:27 |
| **Oligonucleotides and Recombinant DNA** | | |
| Primers for qPCR and genotyping | this paper | Table 1 |
| siRNA Silencer™ Select Negative Control No. 2 siRNA (src2) | Invitrogen | 4390847 |
| siRNA MIF Silencer™ Select | ThermoFisher Scientific | 4390824, ID s8780 |
| siRNA MIF Silencer™ | ThermoFisher Scientific | AM51331, ID 11396 |
| siRNA hCD74 Silencer™ Select | ThermoFisher Scientific | 4392420, ID s2715 |
| siRNA hCD74 Silencer™ Select | ThermoFisher Scientific | 4392420, ID s225179 |
| pcDNA3 GFP | Addgene | 74165 |
| pcDNA3.1/V5-His-TOPO vector | Shi et al., 2006 | PMID: 17045821 |
| pcDNA3.1-CD74 | Shi et al., 2006 | PMID: 17045821 |
| **Software and Algorithms** | | |
| Adobe Photoshop Software | Adobe | https://www.adobe.com/de/creativecloud/plans.html |
| GraphPad Prism | GraphPad | https://www.graphpad.com |
| ImageJ software | Open source | https://imagej.net/Welcome |
| Image Lab™ Software | Biorad | http://www.bio-rad.com/de-de/product/image-lab-software |
| ZEN | Zeiss | https://www.zeiss.de/mikroskopie/produkte/mikroskopsoftware/zen.html |
